# Diversifying Metal–Ligand Cooperative Catalysis in Semi‐Synthetic [Mn]‐Hydrogenases

**DOI:** 10.1002/anie.202100443

**Published:** 2021-05-05

**Authors:** Hui‐Jie Pan, Gangfeng Huang, Matthew D. Wodrich, Farzaneh Fadaei Tirani, Kenichi Ataka, Seigo Shima, Xile Hu

**Affiliations:** ^1^ Laboratory of Inorganic Synthesis and Catalysis Institute of Chemical Sciences and Engineering Ecole Polytechnique Fédérale de Lausanne (EPFL) ISIC-LSCI, BCH 3305 1015 Lausanne Switzerland; ^2^ Max Planck Institute for Terrestrial Microbiology Karl-von-Frisch-Straße 10 35043 Marburg Germany; ^3^ Laboratory for Computational Molecular Design Institute of Chemical Science and Engineering Ecole Polytechnique Fédérale de Lausanne (EPFL) 1015 Lausanne Switzerland; ^4^ Department of Physics Freie Universität Berlin Arnimallee 14 14195 Berlin Germany

**Keywords:** biomimetics, hydrogen activation, hydrogenase, manganese, metal–ligand cooperation

## Abstract

The reconstitution of [Mn]‐hydrogenases using a series of Mn^I^ complexes is described. These complexes are designed to have an internal base or pro‐base that may participate in metal–ligand cooperative catalysis or have no internal base or pro‐base. Only Mn^I^ complexes with an internal base or pro‐base are active for H_2_ activation; only [Mn]‐hydrogenases incorporating such complexes are active for hydrogenase reactions. These results confirm the essential role of metal–ligand cooperation for H_2_ activation by the Mn^I^ complexes alone and by [Mn]‐hydrogenases. Owing to the nature and position of the internal base or pro‐base, the mode of metal–ligand cooperation in two active [Mn]‐hydrogenases is different from that of the native [Fe]‐hydrogenase. One [Mn]‐hydrogenase has the highest specific activity of semi‐synthetic [Mn]‐ and [Fe]‐hydrogenases. This work demonstrates reconstitution of active artificial hydrogenases using synthetic complexes differing greatly from the native active site.

## Graphical abstract

A series of [Mn]‐hydrogenases were reconstituted using specifically designed Mn^I^ complexes. In two catalytically active [Mn]‐hydrogenases, the mode of metal–ligand cooperation is different from that of the native [Fe]‐hydrogenase. One such [Mn]‐hydrogenase exhibits the highest specific activity among all known semi‐synthetic [Mn]‐ and [Fe]‐hydrogenases.

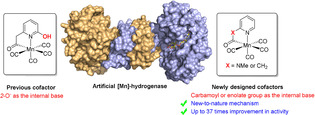

## Introduction

Artificial enzymes incorporating non‐native metal cofactors while exhibiting native activities can serve as important tools for mechanistic studies of enzymes.^[1]^ They also have potential applications in biocatalysis by increasing availability of hard‐to‐access enzymes as well as overcoming substrate specificity.[^1a^, ^2^] Whereas many artificial enzymes have been developed to bring abiological reactivities,^[3]^ only few of them catalyze the native reactions of wild‐type enzymes.[^2^, ^4^]

**Figure 1:**
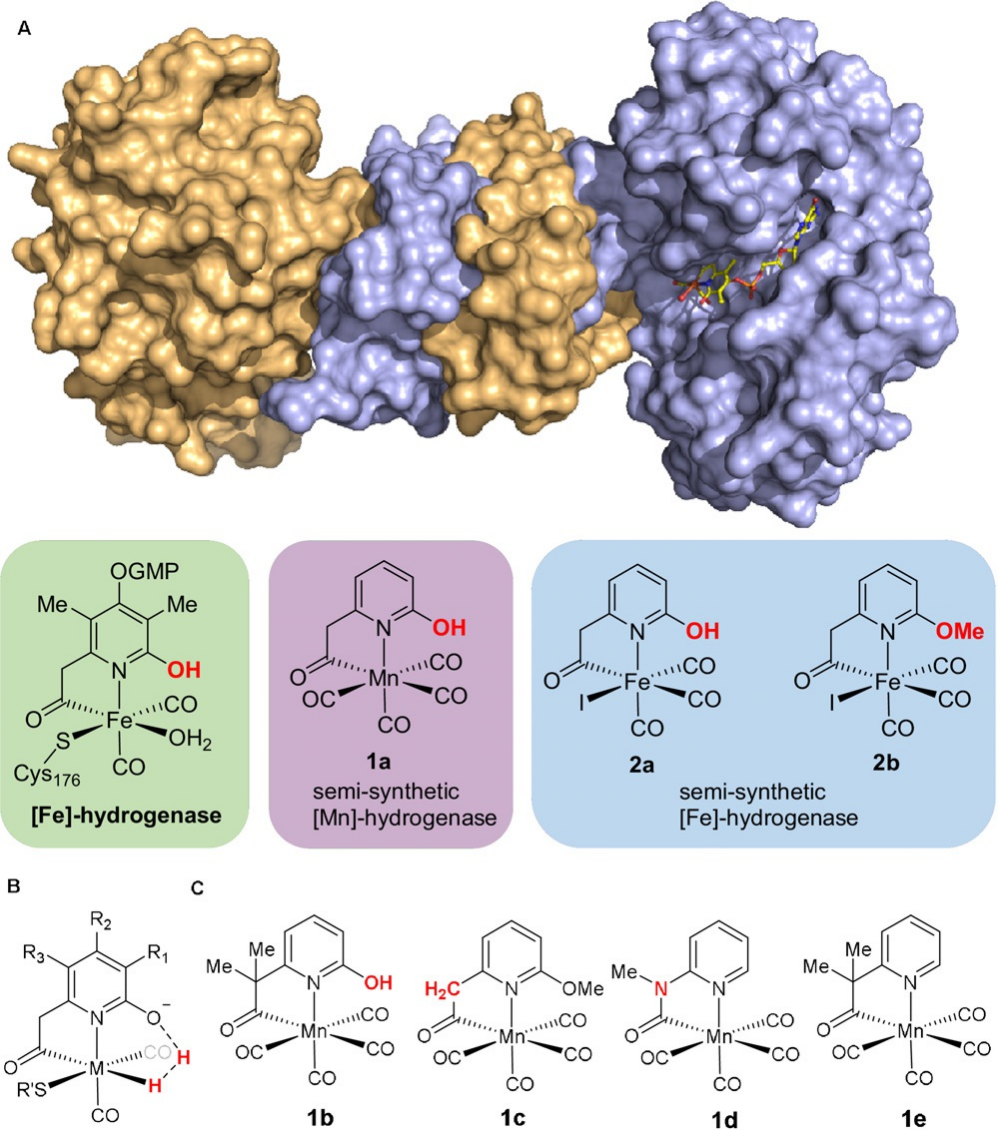
A) Native and semi‐synthetic [Fe]‐ and [Mn]‐hydrogenases, the active site of [Fe]‐hydrogenase, and the synthetic complexes used to reconstitute semi‐synthetic enzymes in the protein free pre‐catalyst forms. B) Metal‐ligand cooperative H_2_ activation in previous [Fe]‐ and [Mn]‐hydrogenases. C) Newly designed Mn complexes for the reconstitution of [Mn]‐hydrogenases. In the reconstitution process, one or two CO ligands bound to the metal complex could probably be substituted or removed.

[Fe]‐hydrogenase catalyzes the hydrogenation of methenyl‐tetrahydromethanopterin (methenyl‐H_4_MPT^+^) to form methylene‐H_4_MPT and its reverse reaction. The forward reaction is a part of the pathways of microbial methanogenesis.^[5]^ [Fe]‐hydrogenase hosts the FeGP cofactor (Figure 1 A) where an Fe^II^ center is coordinated to a pyridone derivative, two *cis*‐CO ligand, a cysteine residue, and a water molecule.^[6]^ We recently reconstituted an active [Mn]‐hydrogenase by incorporating a Mn^I^ mimic of the active site (**1 a**) with the apo‐enzyme of [Fe]‐hydrogenase.^[1d]^ The [Mn]‐hydrogenase exhibited slightly higher specific activity per active metal site than that of a semi‐synthetic [Fe]‐hydrogenase incorporating an Fe^II^ complex analogue (**2 a**).^[1e]^ The synthesis of a catalytically active [Mn]‐hydrogenase paved the way for the current structure–activity study, where the active site is precisely modified via small molecule synthesis. An analogous study using Fe^II^ mimics has been challenging due to their lower stability compared to the Mn^I^ ones.^[7]^

The mechanisms of H_2_ activation in previously reported [Fe]‐ and [Mn]‐hydrogenases all involve metal‐ligand cooperation, where a basic 2‐O^−^ group generated by deprotonation of the 2‐OH group of an pyridone derivative deprotonates the H_2_ molecule coordinated to the metal ion (Figure 1 B).[^1d^, ^1e^, ^6a^] In this study, we design and synthesize a series of Mn^I^ complexes with or without an internal base or pro‐base (Figure 1 C). Among the former complexes, the nature and position of the internal base or pro‐base are different. Only Mn^I^ complexes containing an internal base or pro‐base are active for H_2_ activation. And only such complexes could be used to reconstitute an active [Mn]‐hydrogenases. Based on the nature and position of the internal base or pro‐base of their Mn^I^ cofactors, certain [Mn]‐hydrogenases operate by a non‐native metal‐ligand cooperation mode. One such [Mn]‐hydrogenase exhibits the highest activity among semi‐synthetic [Mn]‐ and [Fe]‐hydrogenases. The work demonstrates the construction of functional hydrogenases using synthetic complexes that operate via a new‐to‐nature reaction mechanism. This strategy might be used to expand the functions of Hmd hydrogenases beyond native reactions using novel substrates other than methenyl‐/methylene‐H_4_MPT and H_2_.

## Results

Subsequent to the work with **1 a**, we designed a series of new Mn^I^ models (**1 b**–**1 e**) that could be used to probe the influence of an internal base for H_2_ activation. Complex **1 b** is an analogue of complex **1 a** in which the two acidic protons on methylene group are replaced by two methyl groups. Like **1 a**, this complex contains a 2‐OH group that can serve as an internal base upon deprotonation. Complex **1 c** has a 2‐OMe group which cannot serve as an internal base, but the methylene moiety at the 6‐position is enolizable and can potentially serve as an internal base upon deprotonation. Complex **1 d** has a carbamoyl group where the tertiary amide moiety can serve as an internal base. Complex **1 e** has no potential basic site and servers as a reference.

**Figure 2:**
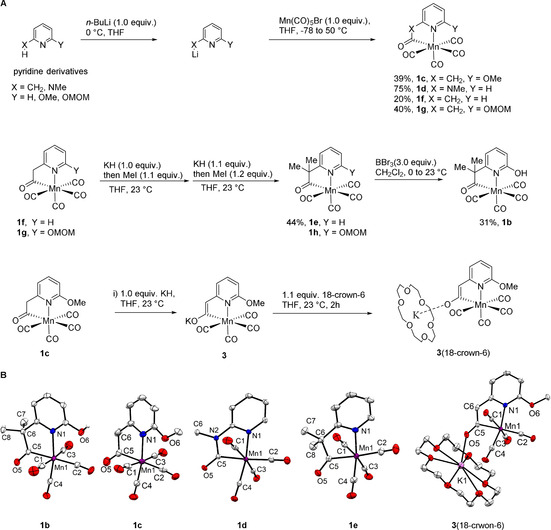
A) Synthesis of Mn model complexes; B) X‐ray structures of **1 b**, **1 c**, **1 d**, **1 e** and **3**(18‐crwon‐6). Thermal ellipsoids are displayed at a 50 % probability.

The synthesis of the Mn complexes is summarized in Figure 2 A. An appropriate pyridine derivate was firstly deprotonated by 1.0 equivalent of *n*‐BuLi to give a lithiated salt, which was directly treated, without purification, with 1.0 equivalent of Mn(CO)_5_Br to yield Mn complexes **1 c**–**1 g**. The methylene groups in **1 f** and **1 g** were methylated twice through a deprotonation/methylation sequence to give complexes **1 e** and **1 h**. Deprotection of the MOM (MOM=methoxymethyl) group in **1 h** using 3.0 equivalents of BBr_3_ afforded complex **1 b**. Deprotonation of the methylene group in **1 c** by reacting with 1.0 equivalent of KH gave complex **3**, which contains an enolate ligand (Figure 2 A). By addition of 1.1 equivalents of 18‐crown‐6 to **3**, **3**(18‐crown‐6) was formed and isolated.

We estimated p*K*_a_ of relevant OH, CH, and amide acids in DMSO using NMR titration experiments (Supporting Information, section 1.4). The p*K*_a_ of the 2‐OH group in **1 a** and **1 b** were 9.0±0.1 and 8.8±0.1, respectively, that of the C(6)‐H moiety in **1 c** was 13.7±0.1, and that of the conjugated acid of **1 d** was between 1.6–3.6.

The IR spectra of **1 a**, **1 b** and **1 c** exhibit four ν(CO) peaks (Supporting Information, Table S1), consistent with the complexes having four CO ligands. In the IR spectra of **1 d** and **1 e**, however, only 3 ν(CO) peaks are observed due to peak overlapping. In the ^13^C NMR spectra of **1 a**–**1 e**, the four CO ligands give only 3 peaks (Supporting Information, Table S2), indicating that the two trans‐orienting CO ligands are equivalent on the NMR scale. Overall, the CO ligands in **1 a**–**1 e** have similar ν(CO) frequencies and ^13^C NMR shifts, indicating a similar electronic property among these complexes. The substituents at the 2‐position of the pyridine ligand have only a minor influence on the electron density at the Mn center. Upon deprotonation of the methylene group in **1 c** to form an enolate group in **3**(18‐crown‐6), the ^1^H NMR chemical shift of the remaining proton on the methylene carbon changed from 3.91 ppm to 5.19 ppm, consistent with the formation of an alkene moiety. This interpretation is also supported by IR spectra, which indicates a more electron‐rich Mn center in **3**(18‐crown‐6) than in **1 c** (Supporting Information, Table S1).

X‐ray crystallography confirms **1 b**–**e** and **3**(18‐crown‐6) as Mn^I^ tetra(carbonyl) complexes ligated by a bidentate N‐C ligand derived from pyridine (Figure 2 B).^[8]^ The coordination geometry of Mn ions is best described as pseudo‐octahedral. The N‐C ligand forms a non‐planar five‐membered metallocycle with the Mn ion. Among the four CO ligands, the one *trans* to the pyridinyl N ligand forms the shortest M‐CO bond (Mn‐C4; Supporting Information, Table S3), consistent with N(pyridine) being the weakest *trans* ligand among N(pyridine), C(acyl), and C(CO). The M–CO distances for the other three CO ligands are rather similar (Supporting Information, Table S3). Mn ion and the ortho‐O atom are confirmed metal–ligand cooperation sites. Their distances in **1 a** and **1 b** are about 3.2 Å. Notably, the distances of the Mn ion to the C6 or N2 atom in **1 c** and **1 d**, respectively, are only about 3.0 Å. This short distance substantiates the possibility of the C6 and N2 atoms serving as an alternate metal‐ligand cooperative site. Upon deprotonation of **1 c** to form **3**(18‐crown‐6), the C5–C6 bond shortened from 1.533(9) Å to 1.396(2) Å, and the C5–O5 bond elongated from 1.216(7) Å to 1.250(2) Å. These changes confirm the presence of a coordinating enolate moiety in **3**(18‐crown‐6).

Only irreversible oxidation and reduction were observed in the cyclic voltammograms of **1 a**–**e** (Supporting Information, Figure S40). Judging from the peak potentials for the oxidation, the electron density of the Mn^I^ ion follows the following order: **1 a**≈**1 b**≈**1 c**>**1 e**>**1 d**.

An H_2_/D_2_ exchange assay was employed to probe the activities of **1 b**–**1 e** and **3**(18‐crown‐6) in H_2_ activation (Table 1; for more information, see the Supporting Information, Section 1.3). The reactions were conducted under a mixed atmosphere of D_2_ (8 bar) and H_2_ (12 bar). The formation of HD indicated H_2_ activation. The initial Mn complexes remained the only identifiable Mn species during and after the reaction; no Mn‐H intermediates were observed. All complexes except **1 e** catalyzed H_2_/D_2_ exchange in the presence of an external base (Table 1). The reactivities have the following order: **1 a**>**1 b**≈**1 c**>**1 d** when *N*‐methylpyrrolidine (MP) was used as the external base (entries 1–3, Table 1). The lower activity of **1 b** compared to **1 a** suggests that the two methyl groups at the C6 position of the ligand hinders H_2_ activation (entries 1 and 2, Table 1). The reactivity of **1 c** depended strongly on the basicity as well as the quantity of the external base. In the presence of one equivalent of a strong base KO^t^Bu (entry 6, Table 1), no H_2_/D_2_ exchange was observed. Increasing the equivalent of KO^t^Bu to 2 equivalents led to a rapid H_2_/D_2_ exchange (entry 7, Table 1). This result could be rationalized by considering that the first equivalent of KO^t^Bu deprotonated the methylene group in **1 c** to form an enolate complex similar to **3**, which could catalyze H_2_/D_2_ exchange only in the presence of an external base (the second equivalent of KO^t^Bu). To probe this hypothesis, **3**(18‐crown‐6) was used as catalyst. Without an external base, no H_2_/D_2_ exchange took place (entry 11, Table 1). With 1.0 equiv of KO^t^Bu, there was still no rapid H_2_/D_2_ exchange (entry 12, Table 1). This result was rationalized by the lack of proton in the system, which is necessary to mediate H/D exchange after H_2_/D_2_ activation. Indeed, when 1.0 equiv of HO^t^Bu was added to the reaction system, fast H_2_/D_2_ exchange was observed (entry 13, Table 1). For **1 c**, the rate of H_2_/D_2_ exchange was much higher in the presence of KO^t^Bu over MP. This rate difference was attributed to an incomplete formation of the enolate complex in the presence of MP. Indeed, the p*K*_a_ of the methylene proton in **1 c** was about 13.7 in DMSO, higher than that of MPH^+^ (10.6). For **1 a**, a similar rate of H_2_/D_2_ exchange was observed in the presence of KO^t^Bu or MP (entries 1 and 14, Table 1). This result could be rationalized considering that the *2*‐OH proton in **1 a** has a p*K*_a_ of 9.0 in DMSO so that both MP and KO^t^Bu are strong enough to fully deprotonate.

**Table 1:** Summary of the results of H_2_/D_2_ exchange experiments.^[a]^ 
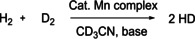

Entry	Complex	Base	*t*	HD:H_2_ ratio	Relative reactivity^[b]^
1	**1 a**	MP	6 h	1:3.3	medium
2	**1 b**	MP	6 h	1:16.8	medium low
3	**1 c**	MP	6 h	1:16.8	medium low
4	**1 d**	MP	6 h	1:43.8	low
5	**1 e**	MP	6 h	<1:100	negligible
6	**1 c**	1.0 equiv KO^t^Bu	6 h	<1:100	negligible
7	**1 c**	2.0 equiv KO^t^Bu	10 min	1:2.2	high
8	**1 c**	Et_3_N	6 h	<1:100	negligible
9	**1 e**	1.0 equiv KO^t^Bu	6 h	<1:100	negligible
10	**1 e**	2.0 equiv KO^t^Bu	6 h	<1:100	negligible
11	**3**(18‐crown‐6)	–	6 h	<1:100	negligible
12	**3**(18‐crown‐6)	1.0 equiv KO^t^Bu	30 min	<1:100	negligible
13	**3**(18‐crown‐6)	1.0 equiv KO^t^Bu + 1.0 equiv HO^t^Bu	30 min	1: 3.2	high
14	**1 a**	2.0 equiv KO^t^Bu	6 h	1: 18.7	medium low

[a] Conditions: D_2_ (8 bar), H_2_ (12 bar), Mn complex 0.03 mmol, CD_3_CN 0.4 mL at ambient temperature; MP=*N*‐methyl pyrollidine, 5.0 equivalent of base relative to the Mn complex. [b] Based on time‐averaged HD:H_2_ ratio determined by ^1^H NMR.

We reconstituted semi‐synthetic [Mn]‐hydrogenases incorporating complexes **1 a**–**1 e** as the metal co‐factors following a previously established protocol.^[1d]^ Due to the potential instability of Mn complexes, the reconstitution experiments were performed as soon as possible after the chemical synthesis (see the Supporting Information). Two equivalents of a Mn complex was dissolved in a methanol solution containing 1 % acetic acid. This solution was then mixed with a solution of one equivalent of the [Fe]‐hydrogenase apoenzyme from *Methanocaldococcus jannaschii* heterologously produced in *Escherichia coli* in the presence of 2 mM guanosine monophosphate (GMP). We reported earlier that external GMP slightly increased the specific activity of semi‐synthetic [Fe]‐hydrogenase and [Mn]‐hydrogenase.[^1d^, ^1e^] The resulting [Mn]‐hydrogenases were named as jHmd (wild)‐**1 a**, jHmd (wild)‐**1 b**, jHmd (wild)‐**1 c**, jHmd (wild)‐**1 d**, jHmd (wild)‐**1 e**, respectively. Mutant enzymes were prepared in the same way but with the His14A and Cys176A apo‐enzymes. They are named as jHmd (H14A)‐**1 a**, jHmd (H14A)‐**1 b**, jHmd (H14A)‐**1 c**, jHmd (H14A)‐**1 d**, jHmd (H14A)‐**1 e**, jHmd (C176A)‐**1 a**, jHmd (C176A)‐**1 b**, jHmd (C176A)‐**1 c**, jHmd (C176A)‐**1 d**, and jHmd (C176A)‐**1 e**.

The activities of [Mn]‐hydrogenases were measured photometrically for both the forward (reduction of methenyl‐H_4_MPT^+^ with H_2_) and the reverse (H_2_ production from methylene‐H_4_MPT) reactions (Table 2). The reconstituted jHmd (wild) enzymes other than jHmd (wild)‐**1 e** exhibited the Hmd activity. In native [Fe]‐hydrogenase, the FeGP cofactor is covalently bound to the protein via the Cys176‐S‐metal coordination. In our previous report as well as this work, a dramatic loss of activity was detected for jHmd (C176A)‐**1 a** (0.06 ± 0.07 U mg^−1^) compared to jHmd (wild)‐**1 a** (7.8±0.7 U mg^−1^). The small residual activity suggests that other interactions such as hydrogen bonding between a metal complex and protein might lead to incorporation of a metal complex in the active site, albeit less efficiently. Additional Cys176Ala mutation studies, however, suggest Cys176‐metal coordination is required for most Mn complexes. Thus, jHmd (C176A)‐**1 b**, jHmd (C176A)‐**1 c**, jHmd (C176A)‐**1 d** and jHmd (C176A)‐**1 e** were totally inactive in both forward and reverse reactions (Table 2, entries 12–15). Based on these properties, we assumed that through reconstitution, the Mn complexes were specifically incorporated into the active site, in which Mn complex coordinated with Cys176‐S. We cannot exclude the possibility that Mn complexes bind at the active site without Cys176S‐Mn bonding though. Infrared spectrum of the reconstituted enzymes were similar to those of the free Mn complexes without protein, which indicated that substantial Mn complex was non‐specifically adsorbed on other parts of the protein (Supporting Information, Figure S41). Previously we fortuitously obtained a sample where the majority of adsorbed Mn complex was incorporated into the active site. The frequency of the two CO bands are similar to those of the native [Fe]‐hydrogenase, which supported the binding mode of the Mn complex to the protein by Cys176‐S‐metal bonding. Based on the intensities of v(CO) band and the amide II band of the protein, we were able to estimate the occupancy of the active site of samples of jHmd (wild)‐**1 a**.^[1d]^ Previous study shown that non‐specifically adsorbed Mn complexes had no contribution to the detected enzymatic activities.^[1d]^

**Table 2:** Occupancy rates and actual specific activities of [Mn]‐hydrogenases.^[a]^ 
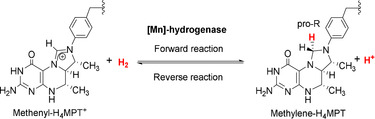

Entry	Samples	Occupancy [%]	Actual specific activity [U mg^−1^]^[b]^ (forward)	Actual specific activity [U mg^−1^] (reverse)
1	jHmd (wild)‐**1 a**	20.3±0.5	7.8± 0.7	1.0± 0.7
2	jHmd (wild)‐**1 b**	70.9±4.6	1.1±0.17	ND
3	jHmd (wild)‐**1 c**	25.8±11.9	37±17	37±16
4	jHmd (wild)‐**1 d**	14.6±2.8	9.3±1.6	ND
5	jHmd (wild)‐**1 e**	58.8±20.5	ND	ND
6	jHmd (H14A)‐**1 a**	–	ND	ND
7	jHmd (H14A)‐**1 b**	–	ND	ND
8	jHmd (H14A)‐**1 c**	–	ND	ND
9	jHmd (H14A)‐**1 d**	–	0.5 ± 0.2^[c]^	ND
10	jHmd (H14A)‐**1 e**	–	ND	ND
11	jHmd (C176A)‐**1 a**	–	0.06± 0.07^[c]^	ND
12	jHmd (C176A)‐**1 b**	–	ND	ND
13	jHmd (C176A)‐**1 c**	–	ND	ND
14	jHmd (C176A)‐**1 d**	–	ND	ND
15	jHmd (C176A)‐**1 e**	–	ND	ND

[a] ND: not detected (the specific activity was less than 0.02 U mg^−1^). [b] The actual specific activities were obtained via dividing the measured specific activities by the active site occupancies. [c] Measured specific activity.

In this study, for [Mn]‐hydrogenases incorporating other Mn complexes, we were not able to obtain samples with only specifically adsorbed complexes. Thus, we developed an alternative and more general method for estimating the occupancy of the active site (Supporting Information, Section 2.2). We assume that jHmd (C176A) cannot bind a Mn complex in the active site as the sulfur ligand from the Cys176 is removed. The v(CO) peaks in reconstituted jHmd (C176A) samples could be attributed to non‐specifically bound Mn complexes. Meanwhile, the v(CO) peaks in reconstituted jHmd (wild) samples were due to both specifically and non‐specifically adsorbed Mn complexes. By subtracting the amount of non‐specifically bound complexes, we obtained the occupancies of the reconstituted jHmd (wild) samples (Scheme S2 for methods and Table 2 and the Supporting Information, Table S5 for results). We acknowledge a substantial level of uncertainty in this analysis due to, for example, a limited spectral resolution or the possibility that jHmd (C176A) and jHmd (wild) would unspecifically bind a different amount of a given Mn complex. Nevertheless, this analysis shall give correct qualitative trends. The occupancy of jHmd (wild) **1 a** obtained by this method (20.3 %) is nearly the same as that estimated by the previous method (20 %).^[1d]^ The occupancies of jHmd (wild) **1 a**, **1 c** and **1 d** were similar (from about 15 % to 25 %). Notably, reconstitution using complexes **1 b** and **1 e** bearing two methyl groups α‐to the acyl donor had much higher occupancies (70.9 % and 58.8 %, respectively).

The measured specific activities were divided by the active site occupancies to give the actual specific activities, which could be compared among different enzymes. [Mn]‐hydrogenases reconstituted with **1 b**–**1 d**, along with **1 a**, were all catalytically active for at least the forward reaction. The [Mn]‐hydrogenase reconstituted with **1 e** was inactive for both forward and reverse reactions.

The activity of jHmd (wild)‐**1 a** determined on samples prepared in this study (7.8± 0.7 for the forward reaction) was nearly identical to that of previously reported sample.^[1d]^ The activity of jHmd (wild)‐**1 b** (1.1±0.17 U mg^−1^ for the forward reaction) was about 14 % of that of jHmd (wild)‐**1 a**, suggesting that the steric hindrance due to the two additional methyl groups at the C6 position of the ligand slows down the reaction. jHmd (wild)‐**1 d**, which contains a tertiary amide basic site, had similar activity (9.3±1.6 U mg^−1^ for the forward reaction) as jHmd (wild)‐**1 a**. The most active enzyme was jHmd (wild)‐**1 c** whose precursor complex has an enolate basic site upon deprotonation. Its actual specific activities for the forward reaction and reverse reaction were 37±17 U mg^−1^ and 37±16 U mg^−1^, which are about 4 and 37 times higher than those of jHmd (wild)‐**1 a**, respectively. The activities of jHmd (wild)‐**1 c** were about 5 times and 8 times higher than those of semi‐synthetic [Fe]‐hydrogenase reconstituted with Fe complex **2 a**.^[1e]^ These activities were about 10 % of those of native [Fe]‐hydrogenase which has the FeGP cofactor. Note that semi‐synthetic [Fe]‐hydrogenase reconstituted with an analogous Fe complex **2 b** (Figure 1 A) was inactive. We attributed the difference in activity to the instability of **2 b** upon deprotonation of the methylene(acyl) group as well as the generally lower activity of Fe mimics compared to Mn mimics.[^7a^, ^9^]

In the native [Fe]‐hydrogenase, His14 is proposed as a proton acceptor from 2‐OH of pyridinol of the FeGP cofactor.^[6a]^ To probe the function of His14 as a base and a part of the proton relay (Supporting Information, Figure S45), we conducted reconstitution studies using His14Ala mutant of the apoenzyme. The resulting [Mn]‐hydrogenases, jHmd (H14A)‐**1 n** (**n**=**a**–**d**) had essentially no detectable activity for both forward and reverse reactions except for jHmd (H14A)‐**1 d** (Table 2). This result suggests that His14 is a necessary base to deprotonate not only 2‐OH but also methylene(acyl) groups to form an internal O^−^ or enolate basic sites in the Mn complexes. In contrast, jHmd (H14A)‐**1 d** retained substantial activity of jHmd (wild)‐**1 d**. This result could be understood by considering that the Mn complex **1 d** has already a basic amide site, and once protonated, the proton on it is rather acidic and can be transferred by H_2_O molecule.

## Discussion

The results in Table 1 indicate that an internal base in a Mn complex is necessary for H_2_ activation. The H_2_/D_2_ exchange was observed only in the presence of an external base because this exchange requires a H^+^/D^+^ exchange in addition to H_2_ activation.

**Figure 3:**
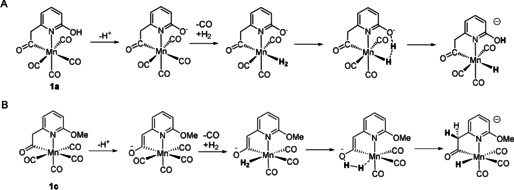
A) Proposed mechanism for H_2_ activation by complexes **1 a** and **1 b**; B) Proposed mechanism for H_2_ activation by complex **1 c**.

It was previously shown, based on DFT computations, that the mechanism of H_2_ activation on **1 a** involved first deprotonation of the 2‐OH group, followed by dissociation of a CO ligand *trans* to the acyl ligand, H_2_ coordination, and then intramolecular heterolytic H_2_ cleavage (Figure 3 A).^[1d]^ A same mechanism is expected for H_2_ activation on **1 b**, but not on **1 c** and **1 d** where the internal bases (C6 or N2) are located at the *6*‐position of the pyridine moiety. For the latter two catalysts, we propose an analogous mechanism. In the case of **1 c** (Figure 3 B), deprotonation of the catalyst gives a coordinated enolate anion. Replacement of a CO *cis* to the acyl ligand by H_2_ then yields a Mn‐H_2_ complex where the H_2_ can be heterolytically cleaved by the cooperative action of the O anion of the enolate and the Mn center. In support of this mechanism, we found significant D incorporation at the methylene position during both H_2_/D_2_ exchange assay and H^+^/D_2_ exchange assay experiments (Supporting Information, Figure S14). A similar mechanism for H_2_ activation was previously proposed for Mn^[10]^ as well as Fe and Ru complexes.^[11]^ Note that deprotonation of methylene(acyl) group by a base was also reported for Fe models of [Fe]‐hydrogenase, but the resulting enolate anion did not have a catalytic role.^[9]^ In the case of **1 d** (Supporting Information, Figure S13), no deprotonation is needed to create a basic site. Thus, the reaction occurs by replacement of a CO by H_2_ followed by heterolytic H_2_ cleavage via a Mn‐O cooperation.

The results in Table 2 indicate a suitable internal base in the Mn complex is required for reconstituting an active [Mn]‐hydrogenase. The results are consistent with the intrinsic activities of Mn complexes (Table 1). Unlike Mn complexes which require an external base to generate the internal base or to mediate H^+^/D^+^ exchange, [Mn]‐hydrogenases require no external base for function, indicating that a basic protein residue exists near the active site. This residue is likely the His14 group which is important for the activity.^[6a]^ The rate of H_2_ activation by a [Mn]‐hydrogenase is much higher than that of the corresponding Mn complex alone (see the Supporting Information, Table S6 for estimated TOFs). The smallest difference was found for jHmd (wild)‐**1 c** and **1 c** (with 2.0 equiv KO^t^Bu, entry 7 Table 1) but the difference is still large; the former has a TOF of about 24 s^−1^, while the latter has a TOF about 0.03 s^−1^. The differences presumably highlight the essential role of protein environment for efficient catalysis.

The reactivity trend of [Mn]‐hydrogenases does not correlate with that of the Mn complexes alone. Different multi‐step reactions and different conditions are involved in enzyme and H_2_/D_2_ exchange assays. The observed reaction rates do not reflect the reactivity towards the same reaction step, i.e., hydrogen activation, because energetics and kinetics of other steps such as deprotonation or hydride transfer also contribute to the overall rate. Interestingly, the most active [Mn]‐hydrogenase contains a Mn complex (**1 c**) that is most active for H_2_/D_2_ exchange under a strong base. Once activated (deprotonated), this complex has the strongest internal base. We hypothesize that a strong internal basic site is beneficial for metal‐ligand cooperative H_2_ activation as long as this basic site is easily accessible in the catalytic cycle. The reactivity trend of [Mn]‐hydrogenases, however, does not linearly correlate with the basicity of internal bases. For example, jHmd (wild)‐**1 a** and jHmd (wild)‐**1 d** have similar activity, but the internal bases at their active sites have quite different basicities. We hypothesize that for these cases the overall reaction barrier does not scale with the barrier for H_2_ activation.

**Figure 4:**
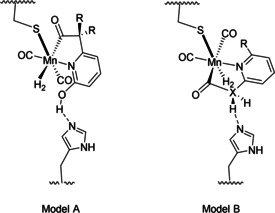
Proposed H_2_ activation models for Mn‐hydrogenases. Model A corresponds for jHmd (wild)‐**1 a** and jHmd (wild)‐**1 b** and Model B corresponds for jHmd (wild)‐**1 c** and jHmd (wild)‐**1 d**.

The mechanism of H_2_ activation by a [Mn]‐hydrogenase is expected to be similar to that of the Mn complex alone. Unlike the native [Fe]‐hydrogenase where the FeGP cofactor contains a GMP group which forms strong interactions with the host protein, consequently the [Mn]‐hydrogenases described here have no GMP group. The Mn cofactor is expected to have more possibilities in orientations in the active site. We envision at least two models. In model A (Figure 4), the acyl ligand of a Mn complex is distal to His14, which is the orientation found for FeGP in the native [Fe]‐hydrogenase. The 2‐OH group is close to His14 so that proton relay from 2‐OH to His14 to form a basic O^−^ group is facile. H_2_ is activated through a mechanism described in Figure 3 A. This model can explain the reactivity of jHmd (wild)‐**1 a** and jHmd (wild)‐**1 b**. In model B (Figure 4), the acyl/carbamoyl ligand of a Mn complex is proximal to His14. Proton relay from X (X=C or N) to His14 as well as from O to X is again facile. H_2_ is activated through a mechanism described in Figure 3 B. This model can explain the reactivity of jHmd (wild)‐**1 c** and jHmd (wild)‐**1 d**. The p*K*_a_ of the 2‐OH group in **1 a** and **1 b**, as well as that of the methylene group in **1 c**, is expected to be much lower in water than in DMSO. As a reference, acetic acid has a p*K*_a_ of 12.3 in DMSO but only 4.8 in water. Thus, proton relay of these groups with His14 in water appears feasible.

The [Fe]‐hydrogenases containing the FeGP cofactor or an iron mimic, as well as jHmd (wild)‐**1 c**, catalyze the forward and reverse reactions in a similar rate.^[1e]^ However, for jHmd (wild)‐**1 a**, jHmd (wild)‐**1 b**, and jHmd (wild)‐**1 d**, the reverse reaction was either much slower than the forward reaction or could not be detected. The reactions were conducted under the same conditions for both [Mn] and [Fe]‐ hydrogenases: the pH for the forward reaction was 7.5 while that for the reverse reaction was pH 6.0. We suspect that the much faster forward reactions for jHmd (wild)‐**1 a**, jHmd (wild)‐**1 b**, and jHmd (wild)‐**1 d** under the employed assay conditions originates from the non‐optimal conditions to generate the internal proton relay for the reverse reactions on jHmd (wild)‐**1 a**, jHmd (wild)‐**1 b**, and jHmd (wild)‐**1 d**. The internal bases in **1 a**, **1 b**, and **1 d** are weaker than that in **1 c**. It is possible that at pH 6.0, the former basic sites are nearly always in the basic forms, i.e., 2‐O^−^ for **1 a** and **1 b** and C(O)N(Me) for **1 d**, which limits the turnover rate for the reverse reaction.

## Conclusion

We have developed a series of Mn^I^ mimics of the active site of [Fe]‐hydrogenase. These complexes are designed to have either a 2‐OH group, an enolizable CH_2_ group, or a tertiary amide group that can serve as an internal base, or no internal base. Only the complexes with internal bases are able to heterolytically cleave H_2_. Incorporation of the Mn complexes into the apo‐enzyme of [Fe]‐hydrogenases result in semi‐synthetic [Mn]‐hydrogenases. The Mn complexes are predicted to bind to the protein via the Cys176 residue. Only the [Mn]‐hydrogenases containing Mn complexes with internal bases are catalytically active for the native reactions of [Fe]‐hydrogenase. H_2_ activation by both Mn complexes alone and [Mn]‐hydrogenases involves metal‐ligand cooperation, where a basic ligand moiety serves as an internal base to deprotonate coordinated H_2_. In addition to the cooperation mode found in [Fe]‐hydrogenase which involves the 2‐O^−^ group of the pyridone ligand of the active site, new cooperation modes involving either an enolate or an amide at the 6‐position of the pyridone ligand as the internal base are operative. Moreover, the [Mn]‐hydrogenase where an enolate moiety serves as the internal base is about 5 times and 8 times more active (forward reaction) than its Mn and Fe counterparts where a 2‐O^−^ group serves as the internal base, respectively. This result represents a good example where a new‐to‐nature reaction mechanism^[12]^ results in substantial activity for the native reaction of an enzyme. The work indicates the possibility to incorporate synthetic complexes distinct from the native active site to reconstitute active Hmd hydrogenases, which can potentially open up new areas of applications such as activation of other small molecules and organic synthesis.

## Conflict of interest

The authors declare no conflict of interest.

## Supporting information

As a service to our authors and readers, this journal provides supporting information supplied by the authors. Such materials are peer reviewed and may be re‐organized for online delivery, but are not copy‐edited or typeset. Technical support issues arising from supporting information (other than missing files) should be addressed to the authors.

Supplementary

## References

[1a] G. Berggren , A. Adamska , C. Lambertz , T. R. Simmons , J. Esselborn , M. Atta , S. Gambarelli , J. M. Mouesca , E. Reijerse , W. Lubitz , T. Happe , V. Artero , M. Fontecave , Nature 2013, 499, 66–69; 23803769 10.1038/nature12239 PMC3793303

[1b] J. Esselborn , C. Lambertz , A. Adamska-Venkatesh , T. Simmons , G. Berggren , J. Noth , J. Siebel , A. Hemschemeier , V. Artero , E. Reijerse , M. Fontecave , W. Lubitz , T. Happe , Nat. Chem. Biol. 2013, 9, 607–609; 23934246 10.1038/nchembio.1311 PMC3795299

[1c] J. F. Siebel , A. Adamska-Venkatesh , K. Weber , S. Rumpel , E. Reijerse , W. Lubitz , Biochemistry 2015, 54, 1474–1483; 25633077 10.1021/bi501391d

[1d] H.-J. Pan , G. Huang , M. D. Wodrich , F. F. Tirani , K. Ataka , S. Shima , X. Hu , Nat. Chem. 2019, 11, 669–675; 31110253 10.1038/s41557-019-0266-1 PMC6591119

[1e] S. Shima , D. Chen , T. Xu , M. D. Wodrich , T. Fujishiro , K. M. Schultz , J. Kahnt , K. Ataka , X. Hu , Nat. Chem. 2015, 7, 995–1002; 26587715 10.1038/nchem.2382

[1f] C. Sommer , C. P. Richers , W. Lubitz , T. B. Rauchfuss , E. J. Reijerse , Angew. Chem. Int. Ed. 2018, 57, 5429–5432; 10.1002/anie.201801914 PMC5924579 29577535

[1g] L. Fruk , C.-H. Kuo , E. Torres , C. M. Niemeyer , Angew. Chem. Int. Ed. 2009, 48, 1550–1574; 10.1002/anie.200803098 19165853

[1h] G. Huang , T. Wagner , U. Ermler , S. Shima , Nat. Rev. Chem. 2020, 4, 213–221. 10.1038/s41570-020-0167-2 37128042

[2] S. Ariyasu , J. K. Stanfield , Y. Aiba , O. Shoji , Curr. Opin. Chem. Biol. 2020, 59, 155–163. 32781431 10.1016/j.cbpa.2020.06.010

[3a] Y.-W. Lin , Coord. Chem. Rev. 2017, 336, 1–27;

[3b] F. Schwizer , Y. Okamoto , T. Heinisch , Y. Gu , M. M. Pellizzoni , V. Lebrun , R. Reuter , V. Köhler , J. C. Lewis , T. R. Ward , Chem. Rev. 2018, 118, 142–231; 28714313 10.1021/acs.chemrev.7b00014

[3c] R. K. Zhang , X. Huang , F. H. Arnold , Curr. Opin. Chem. Biol. 2019, 49, 67–75. 30343008 10.1016/j.cbpa.2018.10.004 PMC6461521

[4a] J. E. Coleman , Nature 1967, 214, 193–194; 4962206 10.1038/214193a0

[4b] P. Cuatrecasas , S. Fuchs , C. B. Anfinsen , J. Biol. Chem. 1967, 242, 1541–1547. 4290246

[5a] S. Shima , U. Ermler , Eur. J. Inorg. Chem. 2011, 963–972;

[5b] L. Bai , T. Fujishiro , G. Huang , J. Koch , A. Takabayashi , M. Yokono , A. Tanaka , T. Xu , X. Hu , U. Ermler , S. Shima , Faraday Discuss. 2017, 198, 37–58; 28294213 10.1039/c6fd00209a

[5c] R. K. Thauer , A. K. Kaster , H. Seedorf , W. Buckel , R. Hedderich , Nat. Rev. Microbiol. 2008, 6, 579–591. 18587410 10.1038/nrmicro1931

[6a] G. Huang , T. Wagner , M. D. Wodrich , K. Ataka , E. Bill , U. Ermler , X. Hu , S. Shima , Nat. Catal. 2019, 2, 537–543;

[6b] S. Shima , O. Pilak , S. Vogt , M. Schick , M. S. Stagni , W. Meyer-Klaucke , E. Warkentin , R. K. Thauer , U. Ermler , Science 2008, 321, 572–575. 18653896 10.1126/science.1158978

[7a] T. Xu , C.-J. M. Yin , M. D. Wodrich , S. Mazza , K. M. Schultz , R. Scopelliti , X. Hu , J. Am. Chem. Soc. 2016, 138, 3270–3273; 26926708 10.1021/jacs.5b12095

[7b] K. M. Schultz , D. Chen , X. Hu , Chem. Asian J. 2013, 8, 1068–1075. 23596151 10.1002/asia.201300232

[8] Deposition Numbers 1981528 (for **1b**), 1981530 (for **1c**), 1981531 (for **1 d**), 1981532 (for **1e**) and 1981533 (for [**3**(18-crwon-6)]) contain the supplementary crystallographic data for this paper. These data are provided free of charge by the joint Cambridge Crystallographic Data Centre and Fachinformationszentrum Karlsruhe Access Structures service www.ccdc.cam.ac.uk/structures.

[9] Y. I. Cho , G. Durgaprasad , M. J. Rose , Inorg. Chem. 2019, 58, 12689–12699. 31497945 10.1021/acs.inorgchem.9b01530

[10a] R. Buhaibeh , O. A. Filippov , A. Bruneau-Voisine , J. Willot , C. Duhayon , D. A. Valyaev , N. Lugan , Y. Canac , J.-B. Sortais , Angew. Chem. Int. Ed. 2019, 58, 6727–6731; 10.1002/anie.201901169 30860308

[10b] S. M. W. Rahaman , D. K. Pandey , O. Rivada-Wheelaghan , A. Dubey , R. R. Fayzullin , J. R. Khusnutdinova , ChemCatChem 2020, 12, 5912–5918.

[11a] C. Gunanathan , D. Milstein , Acc. Chem. Res. 2011, 44, 588–602; 21739968 10.1021/ar2000265

[11b] T. Zell , D. Milstein , Acc. Chem. Res. 2015, 48, 1979–1994. 26079678 10.1021/acs.accounts.5b00027

[12a] P. C. Cirino , F. H. Arnold , Angew. Chem. Int. Ed. 2003, 42, 3299–3301; 10.1002/anie.200351434 12876749

[12b] J. Nazor , S. Dannenmann , R. O. Adjei , Y. B. Fordjour , I. T. Ghampson , M. Blanusa , D. Roccatano , U. Schwaneberg , Protein Eng. Des. Sel. 2007, 21, 29–35; 18093991 10.1093/protein/gzm074

[12c] N.-H. Tran , D. Nguyen , S. Dwaraknath , S. Mahadevan , G. Chavez , A. Nguyen , T. Dao , S. Mullen , T.-A. Nguyen , L. E. Cheruzel , J. Am. Chem. Soc. 2013, 135, 14484–14487. 24040992 10.1021/ja409337v PMC3938315

