# Supplementary material for: Diversifying Metal–Ligand Cooperative Catalysis in Semi‐Synthetic [Mn]‐Hydrogenases
Source: Angew Chem Int Ed Engl. 2021 May 5;60(24):13350–7. doi: 10.1002/anie.202100443 (PMC8251902; doi:10.1002/anie.202100443)
Supplement: Supplementary file 1 — Supplementary [file ANIE-60-13350-s001.pdf]

Angewandte Chemie
Eine Zeitschrift der Gesellschaft Deutscher Chemiker

Supporting Information

Diversifying Metal–Ligand Cooperative Catalysis in Semi-Synthetic [Mn]-Hydrogenases

Hui-Jie Pan, Gangfeng Huang, Matthew D. Wodrich, Farzaneh Fadaei Tirani, Kenichi Ataka, Seigo Shima, and Xile Hu**

anie_202100443_sm_miscellaneous_information.pdf

Contents

1. Supporting information for Mn complexes **3**

1.1 General information 3

1.2 Synthesis of Mn complexes 3

1.3 H_2/D_2 exchange catalyzed by a Mn complex 7

1.4 Procedures for pK_a estimation 8

1.5 Comparison of key characterization data of Mn complexes 13

1.6 Mechanism 14

1.7 Single crystal XRD of Mn complexes 14

1.8 IR spectra of Mn complexes 19

1.9 NMR spectra of Mn complexes 22

1.10 Cyclic voltammetric data of the Mn complexes 29

2. Supporting information for enzymatic study **30**

2.1 IR spectra of [Mn]-hydrogenases and [Fe]-hydrogenase 30

2.2 Calculation methods for occupancy 32

2.3 Enzymatic reactivity and mechanism 35

1. Supporting information for Mn complexes

1.1 General information

A. Chemicals and Reagents

All manipulations were carried out under an N_2(g) atmosphere using glovebox techniques. Solvents were purified using a two-column solid-state purification system (Innovative Technology, NJ, USA) and transferred to the glovebox without exposure to air. Deuterated solvents were purchased from Cambrige Isotope Laboratories, Inc. and Gute Chemie, and were degassed and stored over activated 3Å molecular sieves. All other reagents were purchased from commercial sources. Liquid compounds were degassed by standard freeze-pump-thaw procedures prior to use.

B. Physical Methods

The ^{1}H and ^{13}C spectra were recorded on a Bruker Avance 400 spectrometer. The chemical shifts (δ) are given in parts per million relative to deuterated solvents (THF-d_8: 1.72 ppm in ^{1}H NMR and 25.31 ppm in ^{13}C NMR, CD_3CN: 1.94 ppm in ^{1}H NMR and 118.26 ppm in ^{13}C NMR, DMSO-d_6: 2.50 ppm in ^{1}H NMR). IR spectra of the complexes were recorded on solution samples using a Varian 800 FT-IR spectrometer. Elemental analyses were performed on a Carlo Erba EA 1110 CHN instrument at EPFL. X-ray diffraction studies were carried out in the EPFL Crystallographic Facility. Data collections were performed at low temperature using four-circle kappa diffractometers equipped with CCD detectors. Data were reduced and then corrected for absorption. Solution, refinement and geometrical calculations for all crystal structures were performed by SHELXTL.

1.2 Synthesis of Mn complexes

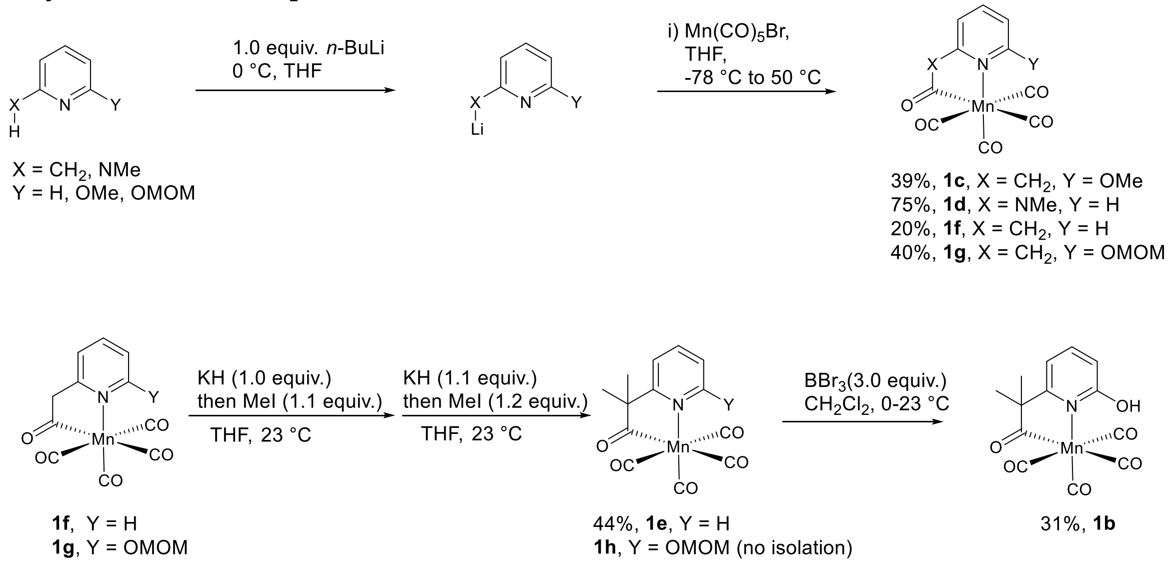

Figure S1. Synthetic routes to Mn complexes

Synthesis of complex 1c

2-methoxyl-*6*-methyl-pyridine (248 mg, 2.0 mmol, 1.0 equiv.) was dissolved in dry THF (12.5 mL) in a Schlenk flask. To this solution was added *n*-BuLi (2.0 mmol, 1.0 equiv.) dropwise at 0 °C. The

solution was further stirred for 30 min at 0 °C. In another Schlenk flask a THF (12.5 mL) solution of $Mn(CO)_5Br$ (548 mg, 2.0 mmol, 1.0 equiv.) was cooled to -78 °C. The solution of deprotonated *2*-methoxyl-*6*-methyl-pyridine was then added dropwise to the $Mn(CO)_5Br$ solution at -78 °C. The resulting mixture was allowed to slowly warm to room temperature and further heated to 50 °C. After stirring at 50 °C overnight, the mixture was cooled to room temperature. The THF solvent was removed. The residue was purified by silica gel chromatography in glovebox using ethyl acetate/hexane as eluent. Single crystal suitable for X-ray diffraction was obtained via layer diffusion of pentane into a THF solution of **1c** at -22 °C. Yield of **1c**: 250 mg, 39%.

^1H NMR (400 MHz, Acetonitrile-d_3) δ 7.85 (t, J = 7.9 Hz, 1H), 7.11 (d, J = 7.4 Hz, 1H), 6.92 (d, J = 8.3 Hz, 1H), 4.04 (s, 3H), 3.91 (s, 2H).

^{13}C NMR (101 MHz, CD_3CN) δ 278.90, 220.48, 215.85, 213.57, 167.36, 162.65, 141.72, 116.18, 105.53, 68.19, 57.16.

HRMS (ESI/QTOF) m/z: $[M + Na]^+$ Calcd for $C_{12}H_8MnNNaO_6^+$ 339.9624; Found 339.9627

IR: ν(cm^{-1}) 1948 (s, terminal CO), 1967 (s, terminal CO), 1983 (s, terminal CO) and 2068 (s, terminal CO)

Anal. Calcd (%) for $C_{12}H_8MnNO_6$: C, 45.5; H, 2.5; N, 4.4. Found: C, 45.9; H, 2.5; N, 4.4.

CCDC-1981530 contain the supplementary crystallographic data for **1c**. These data can be obtained free of charge from *The Cambridge Crystallographic Data Centre via* www.ccdc.cam.ac.uk/data_request/cif.

Synthesis of complex 1d[1]

N-methylpyridin-*2*-amine (432 mg, 4.0 mmol, 1.0 equiv.) was dissolved in dry THF (80 mL) in a Schlenk flask. To this solution was added *n*-BuLi (4.0 mmol, 1.0 equiv.) dropwise at 0 °C. The solution was further stirred for 30 min at 0 °C. In another Schlenk flask a THF (40 mL) solution of $Mn(CO)_5Br$ (1.10 g, 4.0 mmol, 1.0 equiv.) was cooled to -78 °C. The solution of deprotonated *N*-methylpyridin-*2*-amine was then added dropwise to the $Mn(CO)_5Br$ solution at -78 °C. The resulting mixture was allowed to slowly warm to room temperature and further heated to 50 °C. After stirring at 50 °C overnight, the mixture was cooled to room temperature. The THF solvent was removed. The residue was purified by silica gel chromatography in glovebox using ethyl acetate/hexane as eluent. Single crystal suitable for X-ray diffraction was obtained via layer diffusion of pentane into a THF solution of **1d** at -22 °C. Yield of **1d**: 900mg, 75%.

^1H NMR (400 MHz, THF-d_8) δ 8.37 (d, J = 5.7 Hz, 1H), 7.83 (t, J = 8.0 Hz, 1H), 7.03 (d, J = 8.5 Hz, 1H), 6.92 (t, J = 6.5 Hz, 1H), 3.16 (s, 3H).

^{13}C NMR (101 MHz, THF) δ 216.69, 215.71, 214.55, 212.95, 161.11, 153.98, 141.05, 117.64, 109.81, 26.82.

HRMS (APCI/QTOF) m/z: $[M + Na]^+$ Calcd for $C_{11}H_7MnN_2NaO_5^+$ 324.9628; Found 324.9628.

IR: ν(cm^{-1}) 1956 (s, terminal CO), 1983 (s, terminal CO), and 2079 (s, terminal CO)

Anal. Calcd (%) for $C_{11}H_7MnN_2O_5$: C, 43.7; H, 2.3; N, 9.3. Found: C, 44.1; H, 2.5; N, 9.0.

CCDC-1981531 contain the supplementary crystallographic data for **1d**. These data can be obtained free of charge from *The Cambridge Crystallographic Data Centre via* www.ccdc.cam.ac.uk/data_request/cif.

Synthesis of complex 1f

2-methylpyridine (930 mg, 10.0 mmol, 1.0 equiv.) was dissolved in dry THF (50 mL) in a Schlenk flask. To this solution was added *n*-BuLi (10.0 mmol,1.0 equiv.) dropwise at 0 °C. The solution was further stirred for 30 min at 0 °C. In another Schlenk flask a THF (50 mL) solution of $Mn(CO)_5Br$ (2.74 g, 1.0

equiv.) was cooled to -78 °C. The solution of deprotonated *2*-methylpyridine was then added dropwise to the $Mn(CO)_5Br$ solution at -78 °C. The resulting mixture was allowed to slowly warm to room temperature and further heated to 50 °C. After stirring at 50 °C overnight, the mixture was cooled to room temperature. The THF solvent was removed. The residue was purified by silica gel chromatography in glovebox using ethyl acetate/hexane as eluent. Yield of **1f**: 570 mg, 20%.

^{1}H NMR (400 MHz, THF) δ 8.89 (d, *J* = 5.6 Hz, 1H), 7.90 (t, *J* = 7.6 Hz, 1H), 7.55 (d, *J* = 7.8 Hz, 1H), 7.36 (t, *J* = 6.6 Hz, 1H), 3.91 (s, 2H).

^{13}C NMR (101 MHz, THF) δ 270.18, 218.22, 215.40, 214.17, 164.35, 155.39, 139.78, 124.34, 123.77, 67.05.

HRMS (APCI/QTOF) m/z: $[M + Na]^+$ Calcd for $C_{11}H_6MnNNaO_5^+$ 309.9519; Found 309.9513.

Anal. Calcd for $C_{11}H_6MnNO_5$: C, 46.0; H, 2.1; N, 4.9. Found: C, 46.0; H, 2.3; N, 5.0.

Synthesis of complex 1g

2-(methoxymethoxy)-*6*-methylpyridine (612 mg, 4.0 mmol, 1.0 equiv.) was dissolved in dry THF (25 mL) in a Schlenk flask. To this solution was added *n*-BuLi (4.0 mmol, 1.0 equiv.) dropwise at 0 °C. The solution was further stirred for 30 min at 0 °C. In another Schlenk flask a THF (12.5 mL) solution of $Mn(CO)_5Br$ (1.1 g, 4.0 mmol, 1.0 equiv.) was cooled to -78 °C. The solution of deprotonated *2*-(methoxymethoxy)-*6*-methylpyridine was then added dropwise to the $Mn(CO)_5Br$ solution at -78 °C. The resulting mixture was allowed to slowly warm to room temperature and further heated to 50 °C. After stirring at 50 °C overnight, the mixture was cooled to room temperature. The THF solvent was removed. The residue was purified by silica gel chromatography in glovebox using ethyl acetate/hexane as eluent. Yield of **1g**: 550 mg, 40%

^{1}H NMR (400 MHz, CD_3CN) δ 7.82 (t, *J* = 7.9 Hz, 1H), 7.15 (d, *J* = 7.4 Hz, 1H), 7.05 (d, *J* = 8.3 Hz, 1H), 5.46 (s, 2H), 3.93 (s, 2H), 3.54 (s, 3H).

^{13}C NMR (101 MHz, CD_3CN) δ 278.72, 220.50, 216.06, 213.61, 165.53, 162.78, 141.57, 116.97, 107.81, 95.47, 68.34, 57.40.

Anal. Calcd (%) for $C_{13}H_{10}MnNO_7$: C, 45.0; H, 2.9; N, 4.0. Found: C, 45.0; H, 2.9; N, 4.0.

Synthesis of complex 1e

1f (570 mg, 2.0 mmol, 1.0 equiv) was dissolved in dry THF. 1.0 equiv of KH (80 mg) was added slowly to this solution and the mixture was further stirred at 23 °C for 30 min until no gas formation. 1.1 equiv of MeI (156 mg) was then added slowly and the mixture was stirred for 2 h at 23 °C. Then another 1.1 equiv of KH (88 mg) was added slowly and the mixture was further stirred at 23 °C for 30 min until no gas formation. 1.2 equiv of MeI (170 mg) was then added slowly and the mixture was stirred for 2 h at 23 °C. The THF solvent was removed. The residue was purified by silica gel chromatography in glovebox using ethyl acetate/hexane as eluent. Single crystal suitable for X-ray diffraction was obtained via layer diffusion of pentane into a THF solution of **1e** at -22 °C. Yields of **1e**: 280 mg, 44%.

^{1}H NMR (400 MHz, THF-d_8) δ 8.91 (d, *J* = 5.8 Hz, 1H), 7.97 (t, *J* = 7.8 Hz, 1H), 7.57 (d, *J* = 8.0 Hz, 1H), 7.36 (t, *J* = 6.5 Hz, 1H), 1.23 (s, 6H).

^{13}C NMR (101 MHz, THF) δ 274.99, 217.73, 215.23, 214.86, 173.71, 155.05, 140.26, 124.58, 124.11, 70.80, 25.63.

HRMS (APCI/QTOF) m/z: $[M + Na]^+$ Calcd for $C_{13}H_{10}MnNNaO_5^+$ 337.9832; Found 337.9830.

IR: ν(cm^{-1}) 1944 (s, terminal CO), 1967 (s, terminal CO), and 2068 (s, terminal CO)

Anal. Calcd (%) for $C_{13}H_{10}MnNO_5$: C, 49.5; H, 3.2; N, 4.4. Found: C, 49.2; H, 3.0; N, 4.5.

CCDC-1981532 contain the supplementary crystallographic data for **1e**. These data can be obtained free of charge from *The Cambridge Crystallographic Data Centre via* www.ccdc.cam.ac.uk/data_request/cif.

Synthesis of complex 1b

1g (650 mg, 1.87 mmol, 1.0 equiv) was dissolved in dry THF. 1.1 equiv of KH (82 mg) was added slowly to this solution and the mixture was further stirred at 23 °C for 30 min until no gas formation. 1.2 equiv of MeI (319 mg) was then added slowly and the mixture was stirred for 2 h at 23 °C. Then another 1.1 equiv of KH (82 mg) was added slowly and the mixture was further stirred at 23 °C for 30 min until no gas formation. 1.2 equiv of MeI (319 mg) was then added slowly and the mixture was stirred for 2 h at 23 °C. The THF solvent was removed. The residue was filtered through a short silica gel column and concentrated. Formation of **1h** was confirmed by NMR and the crude product was used directly in the next step. Half of the above crude **1h** product was dissolved in 10 mL CH_2Cl_2 at 0 °C and to the mixture 3.0 equiv of BBr_3 in hexane was added dropwise. The mixture was then stirred at 23 °C for 2 h. the reaction was quenched by addition of 10 mL degassed water. The organic layer was collected and the aqueous layer was extracted by CH_2Cl_2 for several times. The combined organic layer was dried by Na_2SO_4 and concentrated. The residue was further purified by silica gel chromatography in glovebox using ethyl acetate/hexane as eluent. Single crystal suitable for X-ray test was obtained via layer diffusion of pentane into a THF solution of **1b** at -22 °C. Yield of **1b**: 95 mg, 31% from **1g**.

^{1}H NMR (400 MHz, THF-d_8) δ 11.47 (s, 1H), 7.73 (t, *J* = 7.8 Hz, 1H), 7.02 (d, *J* = 7.4 Hz, 1H), 6.78 (d, *J* = 8.1 Hz, 1H), 1.19 (s, 6H).

^{13}C NMR (101 MHz, THF) δ 279.12, 219.37, 216.20, 214.94, 172.51, 166.31, 141.30, 115.01, 109.17, 70.57, 26.32.

HRMS (ESI/QTOF) m/z: $[M + H_{-1}]^-$ Calcd for $C_{13}H_9MnNO_6^-$ 329.9816; Found 329.9817.

IR: ν(cm^{-1}) 1949 (s, terminal CO), 1963 (s, terminal CO), 1982 (s, terminal CO) and 2066 (s, terminal CO)

Anal. Calcd (%) for $C_{13}H_{10}MnNO_6$: C, 47.2; H, 3.0; N, 4.2. Found: C, 47.4; H, 3.2; N, 4.2.

CCDC-1981528 contain the supplementary crystallographic data for **1b**. These data can be obtained free of charge from The Cambridge Crystallographic Data Centre via www.ccdc.cam.ac.uk/data_request/cif.

Synthesis of 3(18-crown-6)

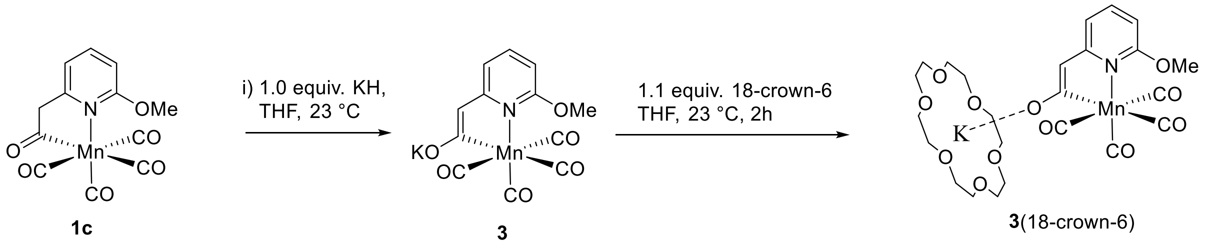

Figure S2. Synthesis of complex **3**(18-crown-6)

To a solution of complex **1c** (200 mg, 0.63 mmol) in THF (5 mL) was added KH (25 mg, 0.63 mmol, 1.0 equiv) slowly under stirring at room temperature. When no more H_2 gas formed, a THF solution of 18-crown-6 (181 mg, 0.69 mmol, 1.1 equiv) was added at once to the mixture. The resulting mixture was further stirred at 23 °C for 2 h before it was filtered through a Teflon membrane to remove all the solid impurity. A layer of Et_2O was then added on top of the THF solution and the mixture was stored at -22 °C. Crystal of **3**(18-crown-6) was obtained in 64% yield (250 mg).

^{1}H NMR (400 MHz, Acetonitrile-d_3) δ 6.89 (t, *J* = 7.9 Hz, 1H), 6.09 (d, *J* = 8.2 Hz, 1H), 5.39 (d, *J* = 7.5 Hz, 1H), 5.19 (s, 1H), 3.75 (s, 3H), 3.57 (s, 24H).

^{13}C NMR (101 MHz, CD_3CN) δ 234.94, 223.05, 222.30, 219.97, 173.58, 166.79, 135.57, 108.73, 105.84, 86.10, 70.88, 55.42.

IR: ν(cm^{-1}) 1890 (s, terminal CO), 1925 (s, terminal CO), 1933 (s, terminal CO) and 2027 (s, terminal CO)

Anal. Calcd (%) for $C_{24}H_{31}MnNO_{12}K$ $(C_4H_8O)_{0.5}$: C, 47.6; H, 5.4; N, 2.1. Found: C, 47.4; H, 5.2; N, 2.3.

CCDC-1981533 contain the supplementary crystallographic data for **3(18-crown-6)**. These data can be obtained free of charge from *The Cambridge Crystallographic Data Centre via* www.ccdc.cam.ac.uk/data_request/cif.

1.3 H_2/D_2 exchange catalyzed by a Mn complex

In a high-pressure NMR tube, a Mn complex (0.03 mmol) and a base were dissolved in 0.4 mL CD_3CN solvent. Then D_2 (8 bar) and H_2 (12 bar, total pressure of mixed gas 20 bar) were added. The reaction was followed by ^{1}H NMR.

During the H_2/D_2 assay, we can only found the peaks of Mn complexes, decomposed ligands and H_2/D_2/HD from ^{1}H NMR. In all cases, no Mn-H species was observed in such assay experiments. As the reaction goes on, the complexes may decompose. Decomposition rates were different from catalyst to catalyst.

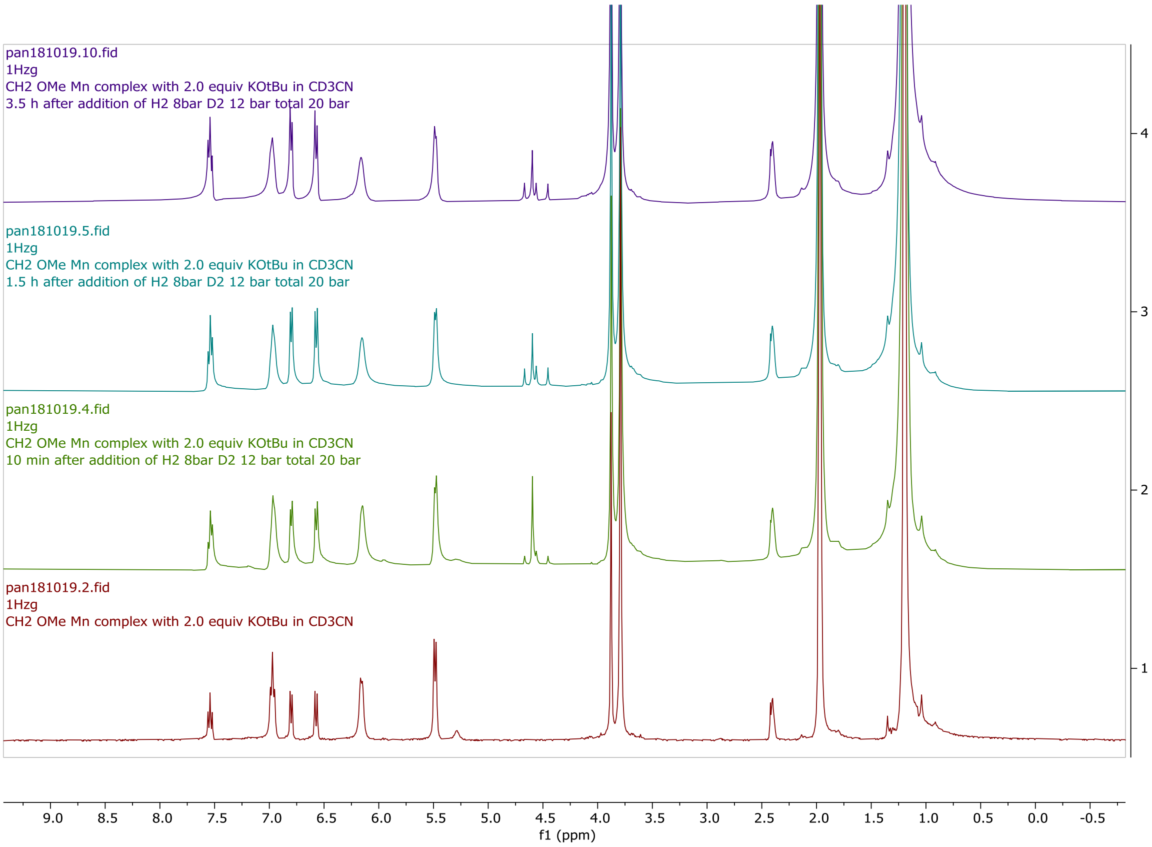

Figure S3 ^{1}H NMR spectra of H_2/D_2 exchange experiment with complex **1c**

1.4 Procedures for pK_a estimation

The pK_a of the corresponding pendant base in a Mn complex was estimated by measuring the equilibrium constant between the base and an acid with a similar pK_a value (Scheme S1). [2] The ratio of each component in this equilibrium was determined by ^{1}H NMR.

$$\text{Mn-B}^- + \text{HA} \rightleftharpoons \text{Mn-BH} + \text{A}^- \quad (1)$$

$$K_e = \frac{[\text{Mn-BH}]\times[\text{A-}]}{[\text{Mn-B}^-]\times[\text{HA}]} = \frac{K_{HA}}{K_{Mn\text{-}BH}} \quad (2)$$

$$K_{Mn\text{-}BH} = \frac{K_{HA}}{K_e} \quad (3)$$

$$pKa_{Mn\text{-}BH} = -\lg K_{Mn\text{-}BH} = -\lg\frac{K_{HA}}{K_e} = pKa_{HA} + \lg K_e \quad (4)$$

Scheme S1. Equations relevant to the estimation of pK_a.

1.4.1. pK_a of complex 1a.

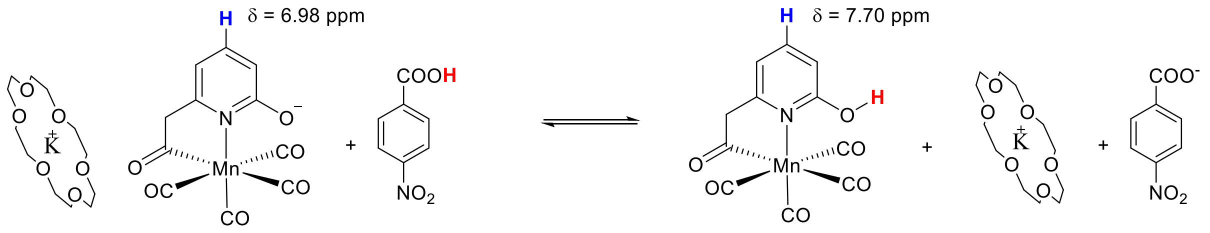

1a(18-crown-6) | *4*-Nitro benzoic acid pKa = 9.1 in DMSO | **1a**

Figure S4. Reaction used for estimating pK_a of complex **1a**

4-nitro benzoic acid was used as the acid and complex **1a**(18-crown-6) was used as base in this case. The pK_a of *4*-nitro benzoic acid in DMSO is 9.1. In an NMR tube, a certain amount of **1a**(18-crown-6) and *4*-niro benzoic acid were dissolved in 0.5 mL of DMSO-d_6. After several minutes when the reaction reached equilibrium, ^{1}H NMR was taken. Since protonation exchange was fast in this reaction, only averaged chemical shifts of complex **1a** and **1a**(18-crown-6) were obtained (shown in Figure S4). The chemical shift of the proton at *4* position of pyridine ligand was used to calculate the ratio of **1a**/**1a**(18-crown-6). For example, when the ratio of **1a**/**1a**(18-crown-6) is 1/1, the resulting chemical shift of *4* position proton in this mixture is 7.34 ppm ((6.98×0.5+7.70×0.5)=7.34).

Test 1: **1a**(18-crown-6) 15.9 mg, 0.0263 mmol, *4*-nitro benzoic acid 2.2 mg, 0.0132 mmol, averaged chemical shift 7.20 ppm. pK_a = 9.0.

Test 2: **1a**(18-crown-6) 15.9 mg, 0.0263 mmol, *4*-nitro benzoic acid 5.5 mg, 0.0329 mmol. averaged chemical shift 7.35 ppm. pK_a = 9.0.

Test 3: **1a**(18-crown-6) 12.8 mg, 0.0212 mmol, *4*-nitro benzoic acid 8.0 mg, 0.0479 mmol. averaged chemical shift 7.42 ppm. pK_a = 8.9.

In summary, pK_a = 9.0 ± 0.1

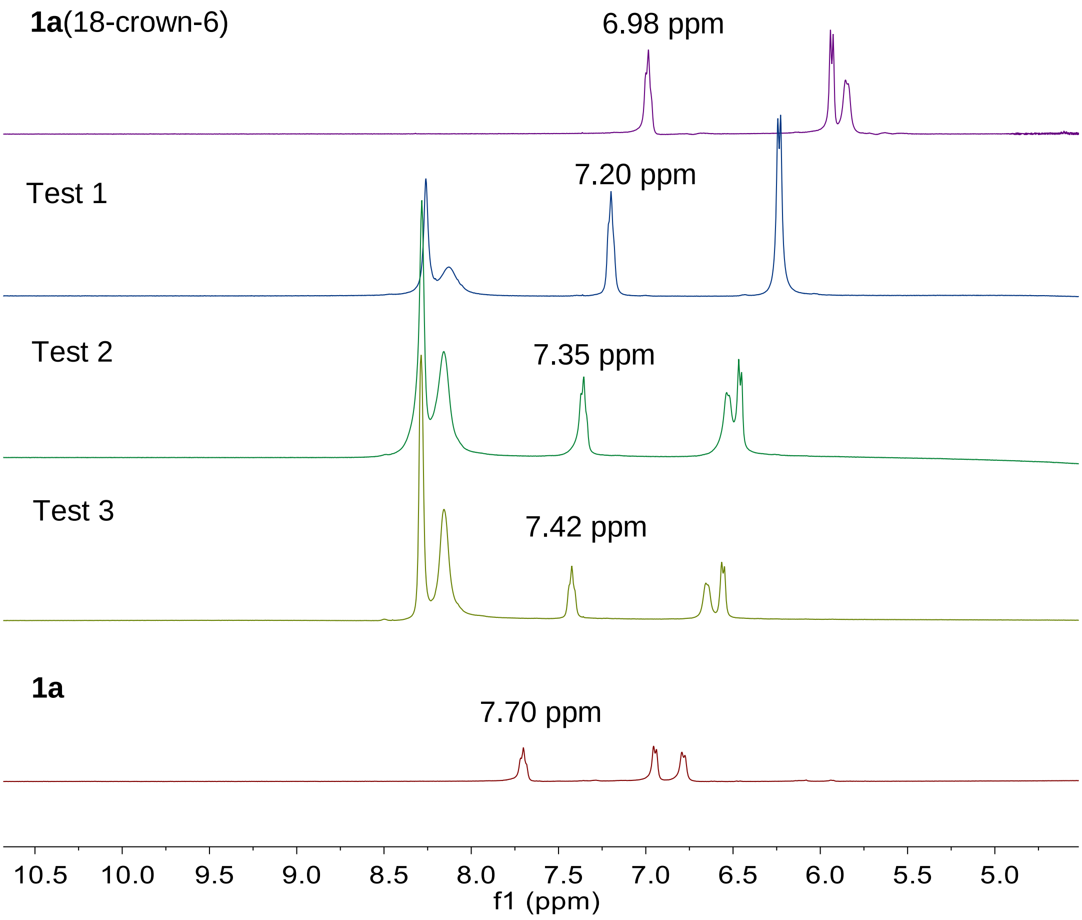

Figure S5. ^{1}H NMR spectra for pK_a estimation reactions of complex **1a**.

1.4.2. pK_a of complex 1b.

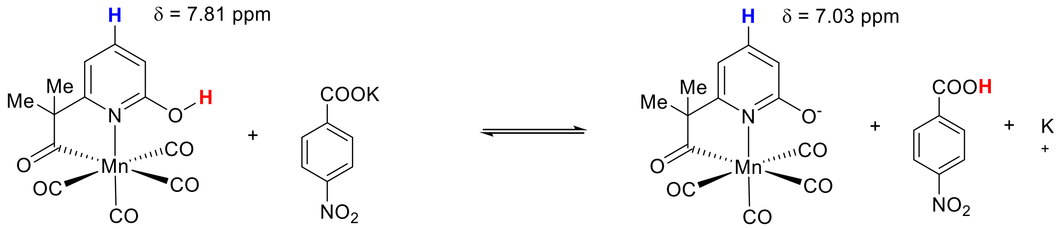

Figure S6. Reaction used for estimating pK_a of complex **1b**

1b was used as the acid and potassium *4*-nitrobenzoate was used as base in this case. The pK_a of *4*-nitro benzoic acid in DMSO is 9.1. In an NMR tube, a certain amount of **1b** and potassium *4*-nitrobenzoate were dissolved in 0.5 mL of DMSO-d_6. After several minutes when the reaction reached equilibrium, ^{1}H NMR was taken. Since protonation exchange was fast in this reaction, only averaged chemical shifts of complex **1b** and **1b**(deprotonated) was obtained (shown in Figure S6). The chemical shift of the proton at *4* position of pyridine ligand was used to calculate the ratio of **1b**/**1b**(deprotonated). For example, when the ratio of **1b**/**1b**(deprotonated) is 1/1, the resulting chemical shift of *4* position proton in this mixture is 7.42 ppm ((7.03×0.5+7.81×0.5)=7.42).

Test 1: **1b** 6.6 mg, 0.020 mmol, potassium *4*-nitrobenzoate 2.0 mg, 0.0098 mmol. Averaged chemical shift 7.52 ppm, pK_a = 8.8.

Test 2: **1b** 6.6 mg, 0.020 mmol, potassium *4*-nitrobenzoate 4.0 mg, 0.0195 mmol. Averaged chemical shift 7.36 ppm, pK_a = 8.8.

Test 3: **1b** 6.6 mg, 0.020 mmol, potassium *4*-nitrobenzoate 6.0 mg, 0.0293 mmol. Averaged chemical shift 7.29 ppm, pK_a = 8.9.

In summary, pK_a = 8.8 ± 0.1

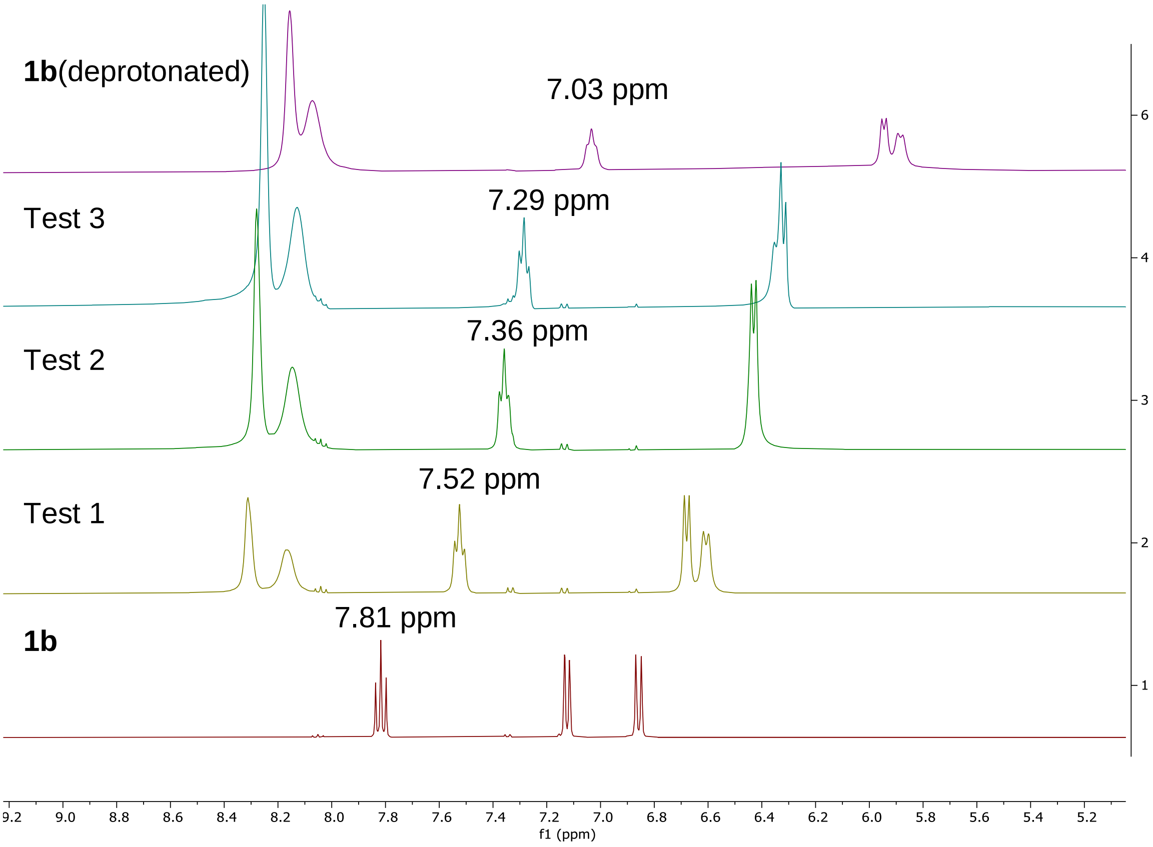

Figure S7. ^{1}H NMR spectra for pK_a estimation reactions of complex **1b**.

1.4.3. pK_a of complex 1c

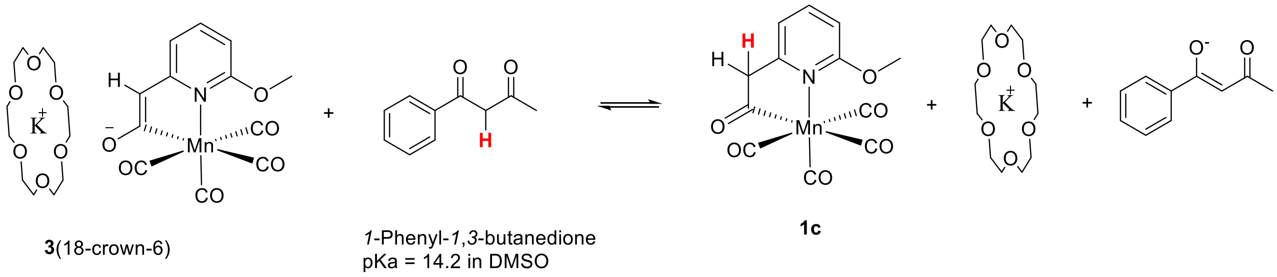

Figure S8. Reaction used for estimating pK_a of complex **1c**

1-Phenyl-*1*,*3*-butanedione was used as the acid and **3**(18-crown-6) was used as base in this case. The pK_a of *1*-phenyl-*1*,*3*-butanedione in DMSO is 14.2. In an NMR tube, certain amount of **3**(18-crown-6) and *1*-Phenyl-*1*,*3*-butanedione were dissolved in 0.5 mL of DMSO-d_6. After several minutes when the reaction reached equilibrium, ^{1}H NMR was taken. In an NMR spectrum, there were two set of

complexes peaks corresponding to **3**(18-crown-6) and **1c** (Figure S8). Integration of these two set of peaks gave the ratio of **3**(18-crown-6)/**1c**.

Test 1: **3**(18-crown-6) 17.5 mg, 0.0283 mmol, *1*-Phenyl-*1*,*3*-butanedione 2.0 mg, 0.0123 mmol. Calculated pK_a = 13.6.

Test 2: **3**(18-crown-6) 17.5 mg, 0.0283 mmol, *1*-Phenyl-*1*,*3*-butanedione 4.0 mg, 0.0246 mmol. Calculated pK_a = 13.7.

In summary, pK_a = 13.7 ± 0.1

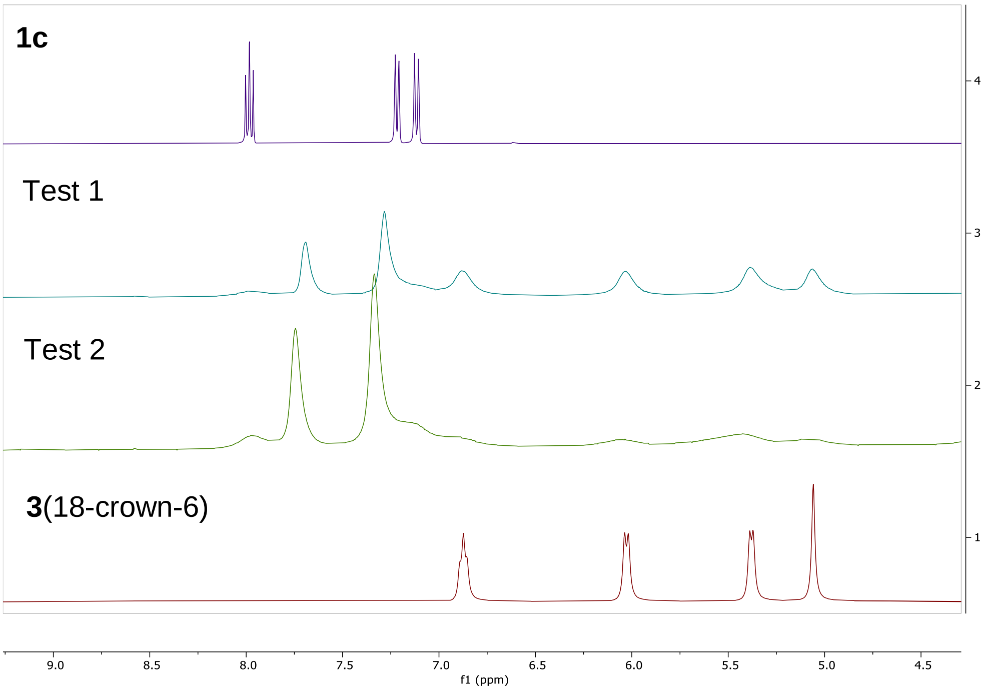

Figure S9. ^{1}H NMR spectra for pK_a estimation reactions of complex **1c**.

1.4.4. pK_a of complex 1d

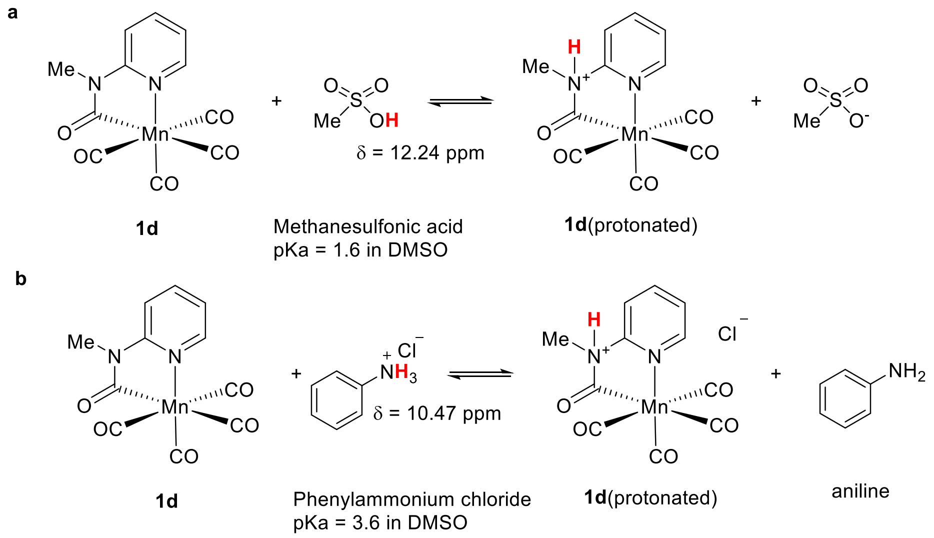

Figure S10. Reactions used for estimating pK_a of complex **1d**

Methanesulfonic acid and phenylammonium chloride were used as the acid and **1d** was used as the base in this case. The pK_a of methanesulfonic acid and phenylammonium chloride in DMSO are 1.6 and 3.6, respectively. In an NMR tube, certain amount of **1d** and methanesulfonic acid or phenylammonium chloride were dissolved in 0.5 mL of DMSO-d_6. After several minutes when the reaction reached equilibrium, ^{1}H NMR was taken.

Test 1: **1d** 21.7 mg, 0.0719 mmol, Methanesulfonic acid 2.4 mg, 0.0250 mmol. Averaged chemical shift of acid proton was 6.05 ppm.

Test 2: **1d** 11.4 mg, 0.0377 mmol, Methanesulfonic acid 2.4 mg, 0.0250 mmol. Averaged chemical shift of acid proton was 6.98 ppm.

As the chemical shift of acid proton greatly changed after addition of **1d** compared to that of methanesulfonic acid, we can expect a weaker acidity of **1d**(protonated) than methanesulfonic acid. Therefore the pK_a of **1d**(protonated) is larger than 1.6 in DMSO.

Test 3: **1d** 10.9 mg, 0.0361 mmol, Phenylammonium chloride 4.5 mg, 0.0349 mmol. Averaged chemical shift of acid proton was 10.24 ppm.

Test 4: **1d** 23.9 mg, 0.0791 mmol, Phenylammonium chloride 2.4 mg, 0.0349 mmol. Averaged chemical shift of acid proton was 10.21 ppm.

The chemical shift of acid proton only had slight changes after addition of excess amount of **1d**. we can expect a stronger acidity of **1d**(protonated) than phenylammonium chloride. Therefore the pK_a of **1d**(protonated) is smaller than 3.6 in DMSO.

In summary, the pK_a of **1d**(protonated) should lie between 1.6-3.6 in DMSO.

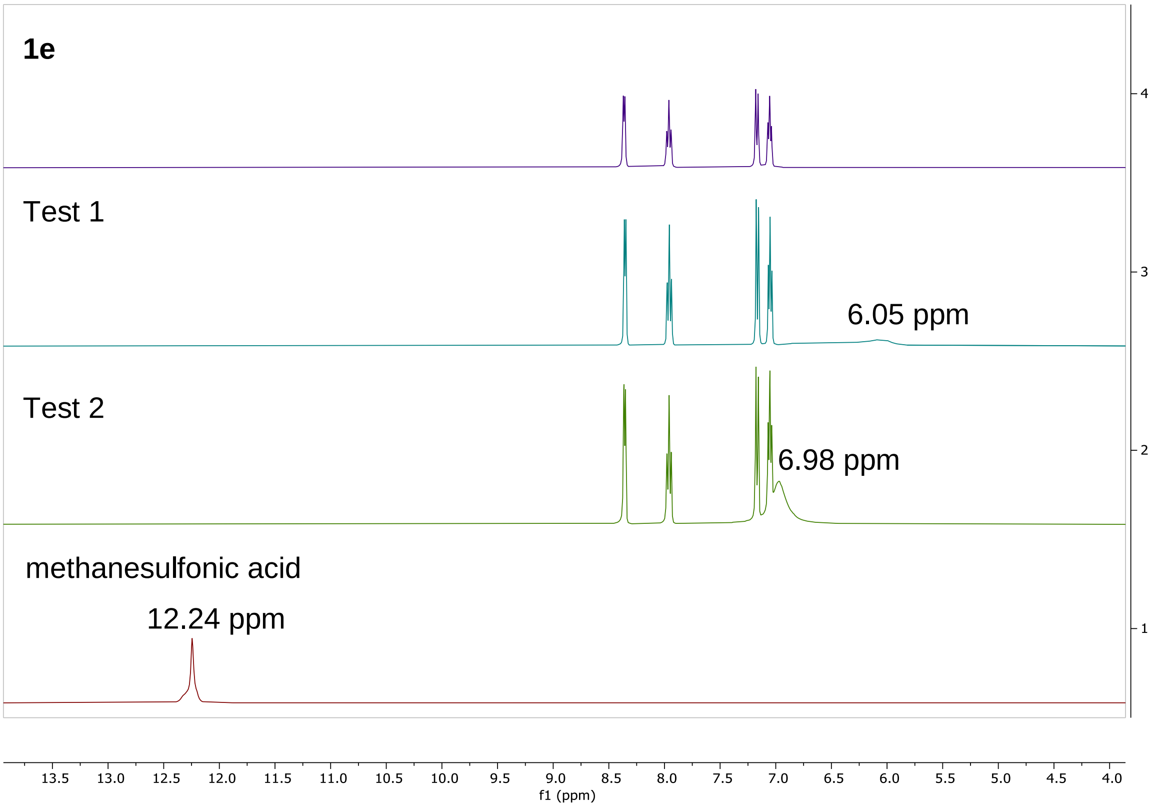

Figure S11. ^{1}H NMR spectra for pK_a estimation reactions of complex **1d** with methanesulfonic acid

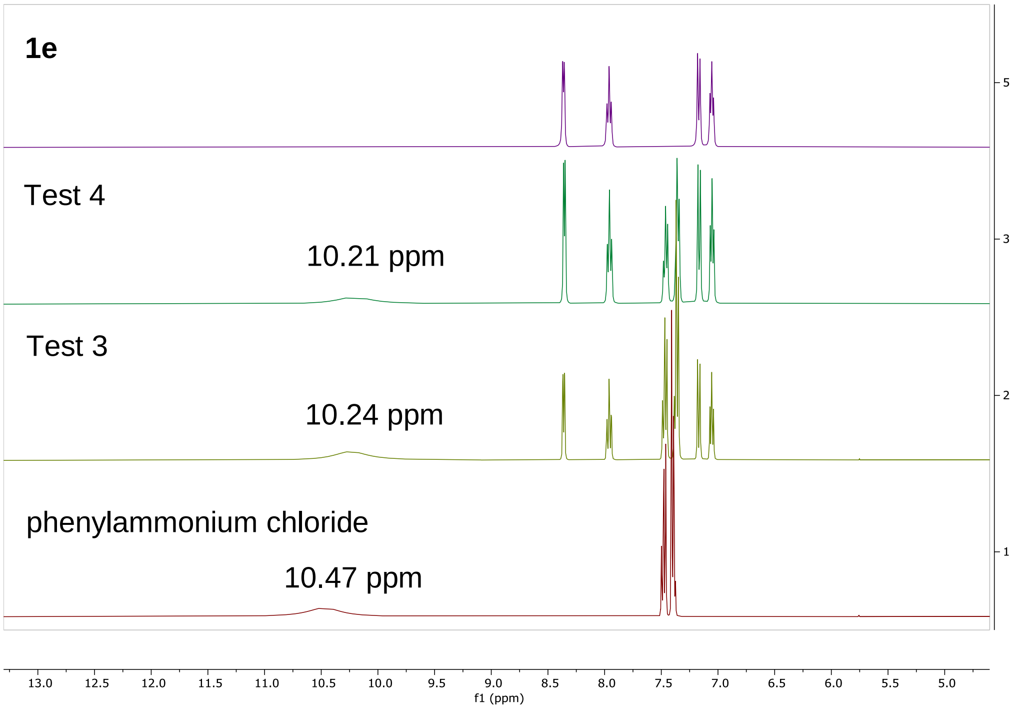

Figure S12. ^{1}H NMR spectra for pK_a estimation reactions of complex **1d** with phenylammonium chloride

.

1.5 Comparison of key characterization data of Mn complexes

Table S1. Selected infrared data

Entry	complex	CO in IR
1	**1a**	2068, 1983, 1967, 1948
2	**1b**	2066, 1982, 1963, 1949
3	**1c**	2068, 1983, 1967, 1948
4	**1d**	2079, 1983, 1956
5	**1e**	2068, 1967, 1944
6	**3**(18-crwon-6)	2027, 1933, 1925, 1890
7[3]	**1a**(18-crwon-6)	2052, 1972, 1944, 1901

Table S2. Selected ^{13}C NMR data.

Entry	complex	CO in ^{13}C NMR	Acyl in ^{13}C NMR
1	**1a**	220.3, 215.9, 213.6	280.8
2	**1b**	219.4, 216.2, 214.9	279.1
3	**1c**	220.5, 215.9, 213.6	278.9
4	**1d**	216.7, 214.6, 213.0	215.7
5	**1e**	217.7, 215.2, 214.9	275.0
6	**3**(18-crwon-6)	223.1, 222.3, 220.0	234.9
7	**1a**(18-crwon-6)	220.9, 219.4, 216.2,	277.2

Table S3. Selected X-ray structural data

Entry	complex	Mn-N (Å)	Mn-C5 (Å)	Mn-C1 (Å)	Mn-C2 (Å)	Mn-C3 (Å)	Mn-C4 (Å)	Mn-X (Å)	Mn-Y (Å)
1	**1a**	2.084	1.999	1.847	1.864	1.857	1.805	2.944	3.173
2	**1b**	2.085	2.007	1.845	1.869	1.868	1.794	3.008	3.186
3	**1c**	2.110	2.033	1.864	1.863	1.865	1.792	2.972	3.190
4	**1d**	2.043	2.028	1.863	1.848	1.855	1.816	2.867	--
5	**1e**	2.065	2.028	1.864	1.858	1.850	1.813	3.021	--
6	**3**(18-crwon-6)	2.084	2.125	1.834	1.836	1.834	1.784	2.909	3.184
7	**1a**(18-crwon-6)	2.068	2.036	1.818	1.852	1.847	1.792	2.963	3.227

1.6 Mechanism

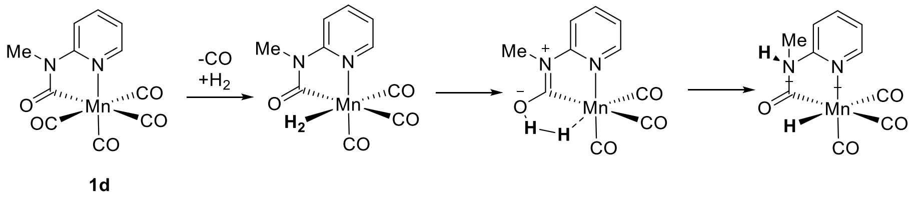

Figure S13. Proposed H_2 activation mechanism for complex **1d**

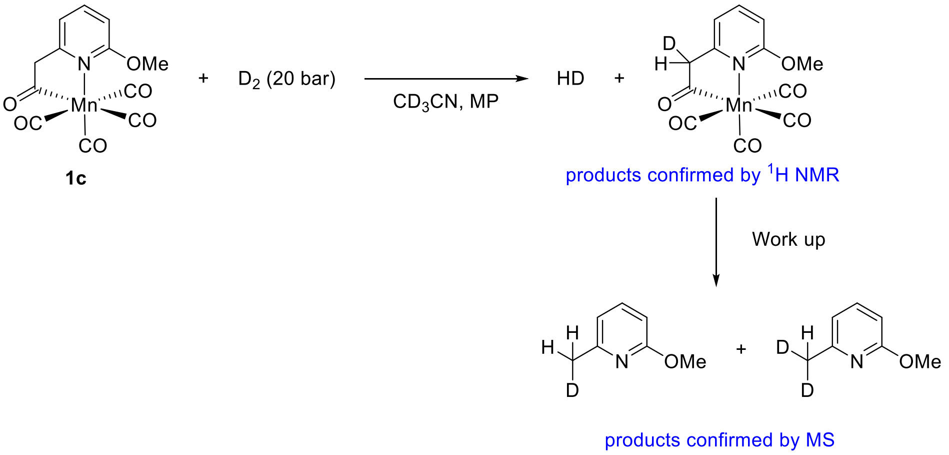

Figure S14. H^+/D_2 exchange assay confirmed the involvement of methylene(acyl) moiety in H_2 activation process

1.7 Single crystal XRD of Mn complexes

Crystal Growth

Single yellow crystals of complex **1b** were obtained by recrystallisation from THF/Hexane at -22 °C. Single yellow crystals of complex **1c** were obtained by recrystallisation from THF/pentane at -22 °C. Single colourless needle-shaped crystals of complex **1d** and **1e** were obtained by recrystallisation from THF/Hexane at -22 °C. Single yellow prism-shaped crystals of **3(18-crown-6)** were obtained by recrystallisation from THF/Et_2O at -22 °C.

Data Collections of 1b, 1d and 3(18-crown-6)

Bragg-intensities of **1b**, **1d** and **3(18-crown-6)** were collected at low temperature using Cu*Kα* radiation. A Rigaku SuperNova dual system diffractometer with an Atlas CCD detector was used for compound **1b**, and one equipped with an Atlas S2 CCD detector for compounds **1d** and **3(18-crown-6)**. The datasets were reduced and corrected for absorption, with the help of a set of faces enclosing the crystals as snugly as possible, with *CrysAlis^{Pro}* [4].

Data Collections of 1c and 1e

Bragg intensities of **1c** and **1e** were measured at low temperature and using Mo*K*α radiation, on a Bruker *APEX II CCD* diffractometer equipped with a κ-geometry goniometer. The datasets were reduced by *EvalCCD* [5] and then corrected for absorption [6].

The solutions and refinements of the structures were performed by the latest available version of *ShelXT* [7] and *ShelXL* [8] using *Olex2* [9] as the graphical interface. All non-hydrogen atoms were refined anisotropically using full-matrix least-squares based on $|F|^2$. The hydrogen atoms in **1b**, **1c** and **3(18-crown-6)** were placed at calculated positions by means of the "riding" model where each H-atom was assigned a fixed isotropic displacement parameter with a value equal to 1.2 U_{eq} of its parent C-atom (1.5 U_{eq} for the methyl groups), but the hydrogen atom bound to O6 in **1b** and all hydrogen atom positions in **1d** and **1e** were found in a difference map and refined freely.

Crystallographic and refinement data are summarized in **Table S4**. The CCDC numbers 1981528 and 1981530-1981533 for compounds **1b, 1c, 1d, 1e and 3(18-crown-6)** contain the supplementary crystallographic data for this paper. These data can be obtained free of charge via www.ccdc.cam.ac.uk/data_request/cif.

Table S4. Crystallographic data of **1b**, **1c**, **1d**, **1e** and **3(18-crown-6)**.

**Compound**	**1b**	**1c**	**1d**	**1e**	**3(18-crown-6)**
Formula	$C_{13}H_{10}MnNO_6$	$C_{12}H_8MnNO_6$	$C_{11}H_7MnN_2O_5$	$C_{13}H_{10}MnNO_5$	$C_{26}H_{35}KMnNO_{12.5}$
$D_{calc.}$/ g cm^{-3}	1.562	1.640	1.658	1.553	1.438
μ/mm^{-1}	7.887	1.052	9.073	0.996	5.330
Formula Weight	331.16	317.13	302.13	315.16	655.59
Colour	clear pale yellow	yellow	colourless	colourless	clear dark yellow
Shape	prism	irregular	needle	prism	prism
Size/mm^3	0.50×0.38×0.28	0.30×0.25×0.20	0.91×0.16×0.07	0.46×0.36×0.20	0.29×0.20×0.06
T/K	140.00(10)	140(2)	100.01(10)	120(2)	100.00(10)
Crystal System	monoclinic	trigonal	monoclinic	monoclinic	triclinic
Flack Parameter		0.11(3)			
Space Group	$P2_1/c$	$P3_1$	$P2_1/c$	$P2_1/c$	$P\bar{1}$
a/Å	10.4076(3)	10.736(3)	7.00671(13)	8.4455(7)	9.2173(3)
b/Å	16.8623(3)	10.736(3)	21.0613(3)	12.7802(7)	13.4904(4)
c/Å	16.6523(3)	9.6512(13)	8.77555(17)	12.8839(17)	13.7905(4)
α/°	90	90	90	90	63.419(3)
β/°	105.442(2)	90	110.797(2)	104.211(8)	88.255(2)
γ/°	90	120	90	90	81.097(2)
V/Å^3	2816.92(10)	963.4(5)	1210.63(4)	1348.1(2)	1513.58(8)
Z	8	3	4	4	2
Z'	2	1	1	1	1
Wavelength/Å	1.54184	0.71073	1.54184	0.71073	1.54184
Radiation type	Cu$K\alpha$	Mo$K\alpha$	Cu$K\alpha$	Mo$K\alpha$	Cu$K\alpha$
Θ_{min}/°	3.802	3.042	4.198	3.581	3.587
Θ_{max}/°	73.602	25.290	76.053	34.999	76.183
Measured Refl's.	19527	9491	7791	29755	13709
Ind't Refl's	5603	2328	2468	5879	6241
Refl's with I > 2σ(I)	5196	2140	2370	5048	6042
R_{int}	0.0497	0.0671	0.0224	0.0203	0.0197
Parameters	391	183	201	221	398
Restraints	0	1	0	0	39
Largest Peak/e Å^{-3}	0.559	0.381	0.307	0.458	0.458
Deepest Hole/e Å^{-3}	-0.591	-0.306	-0.260	-0.576	-0.414
GooF	1.089	1.142	1.052	1.145	1.025
wR_2 (all data)	0.1100	0.0861	0.0616	0.0712	0.0764
wR_2	0.1081	0.0816	0.0611	0.0629	0.0756
R_1 (all data)	0.0452	0.0440	0.0231	0.0370	0.0313
R_1	0.0420	0.0369	0.0223	0.0263	0.0301
CCDC number	1981528	1981530	1981531	1981532	1981533

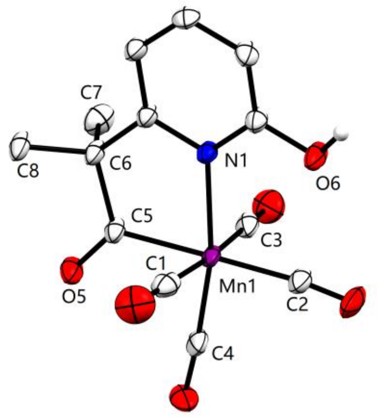

Figure S15. X-ray structure of complex **1b**. Thermal ellipsoids are displayed at a 50% probability. CCDC-1981528.

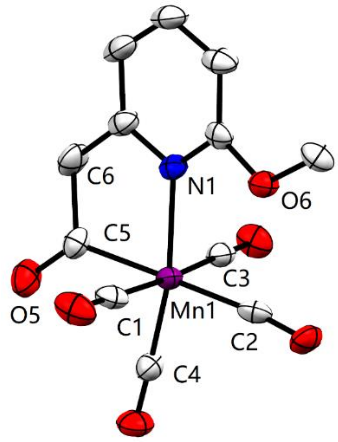

Figure S16. X-ray structure of complex **1c**. Thermal ellipsoids are displayed at a 50% probability. CCDC-1981530.

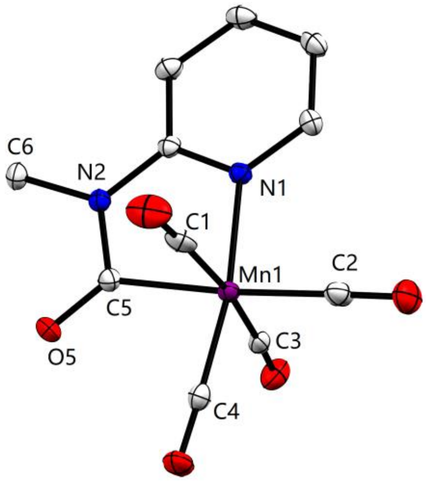

Figure S17. X-ray structure of complex **1d**. Thermal ellipsoids are displayed at a 50% probability. CCDC-1981531.

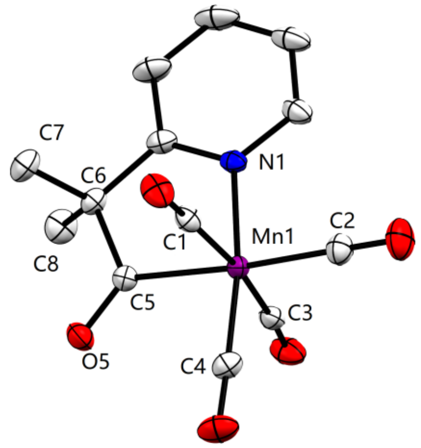

Figure S18. X-ray structure of complex **1e**. Thermal ellipsoids are displayed at a 50% probability. CCDC-1981532.

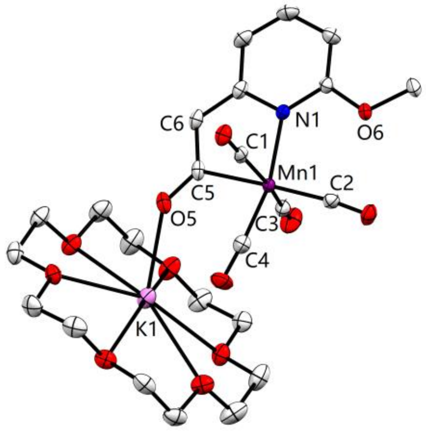

Figure S19. X-ray structure of complex **3**(18-crown-6). Thermal ellipsoids are displayed at a 50% probability. CCDC-1981533.

1.8 IR spectra of Mn complexes

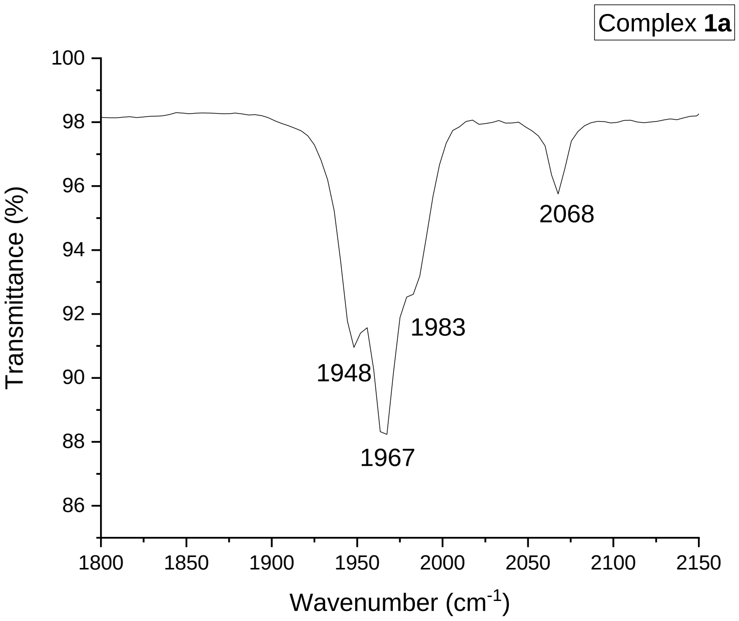

Figure S20. IR spectrum of complex **1a**[3]

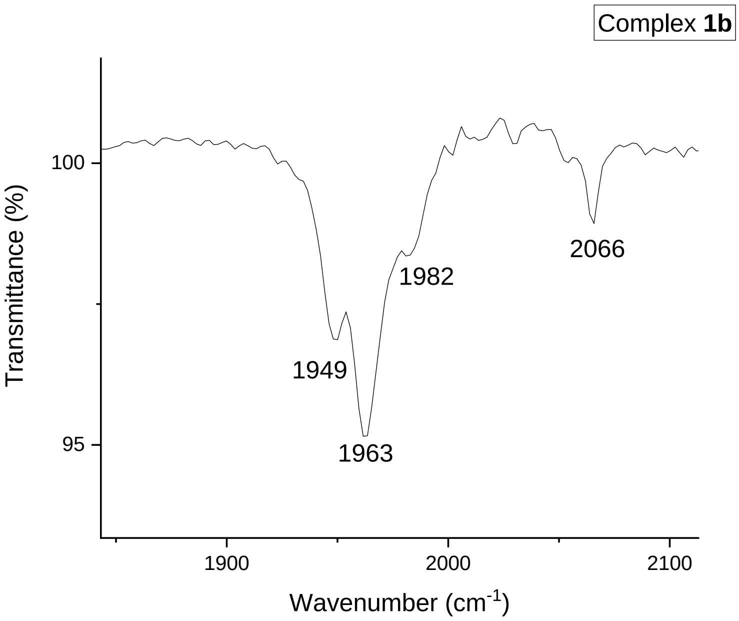

Figure S21. IR spectrum of complex **1b**

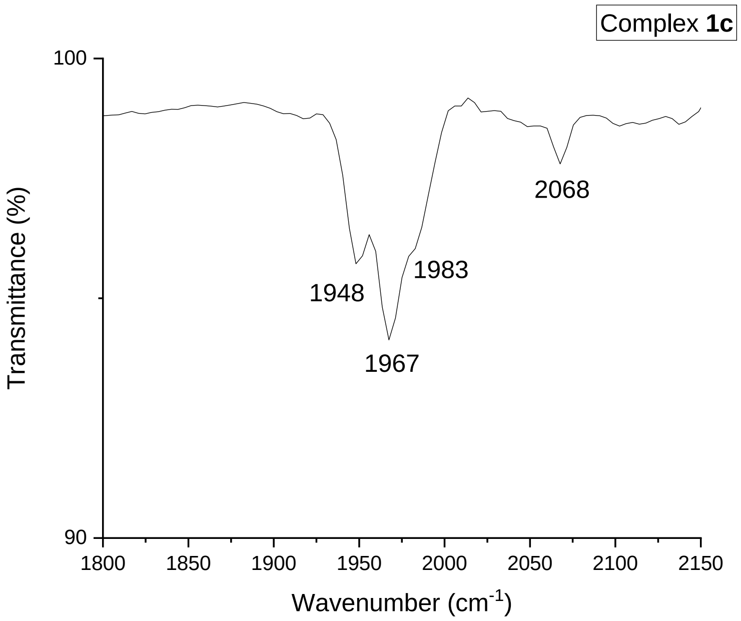

Figure S22. IR spectrum of complex **1c**

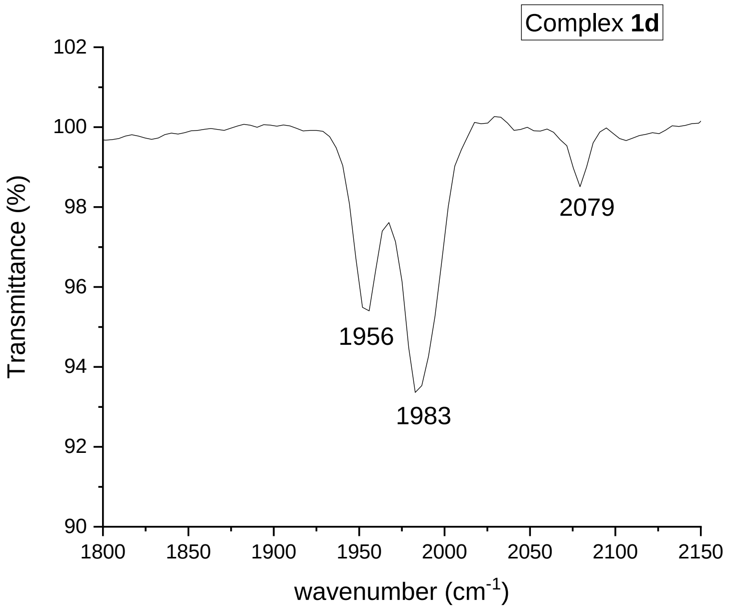

Figure S23. IR spectrum of complex **1d**

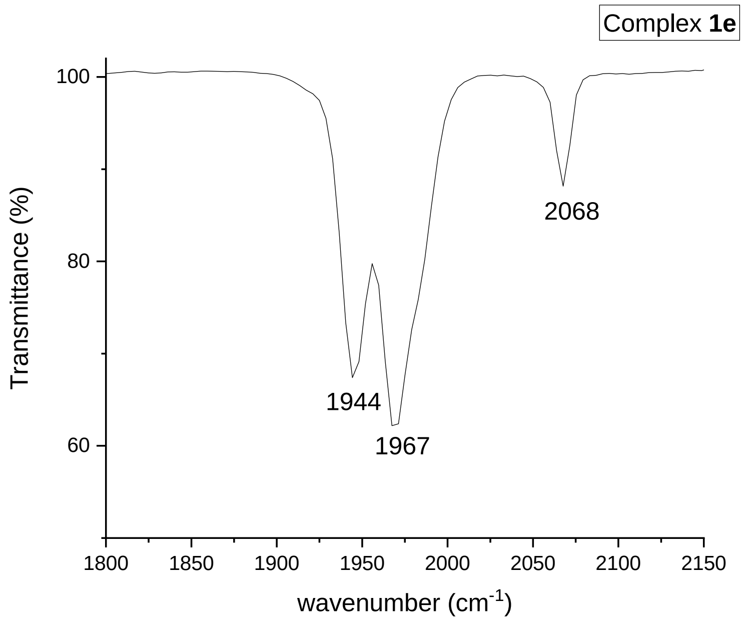

Figure S24. IR spectrum of complex **1e**

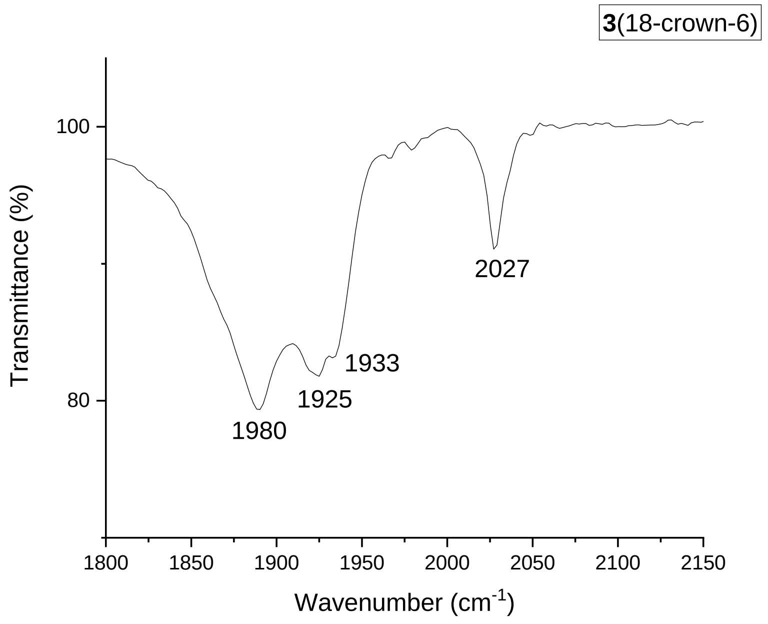

Figure S25. IR spectrum of complex **3**(18-crown-6)

1.9 NMR spectra of Mn complexes

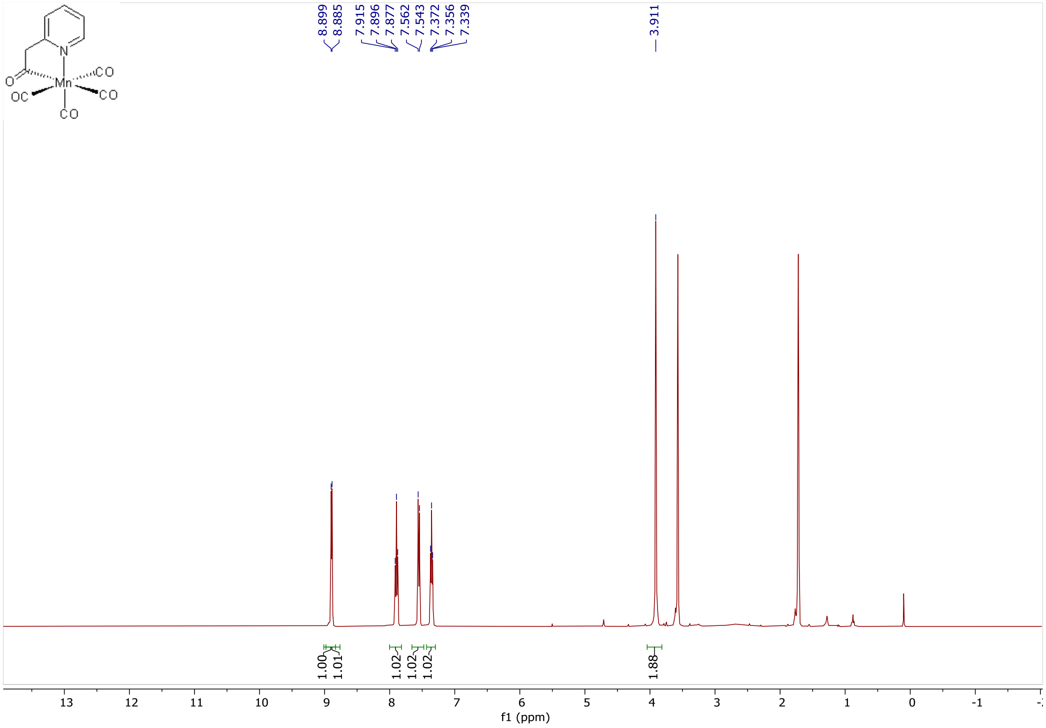

Figure S26. ^{1}H NMR spectrum of complex **1f**

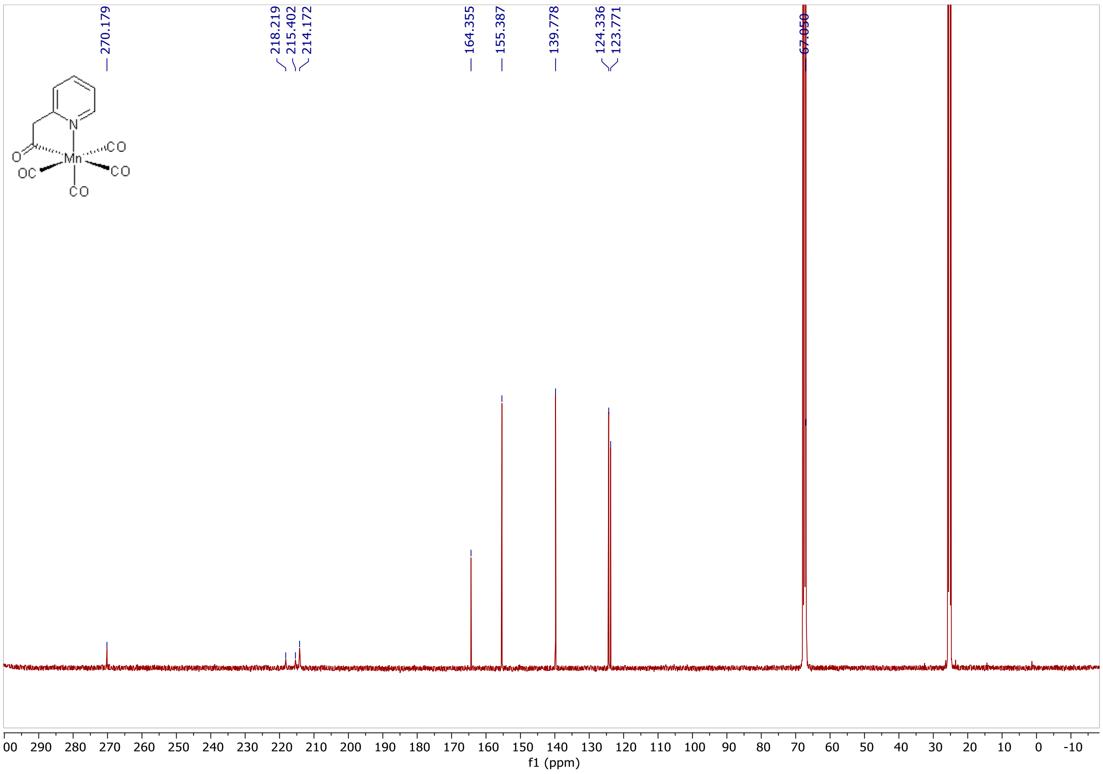

Figure S27. ^{13}C NMR spectrum of complex **1f**

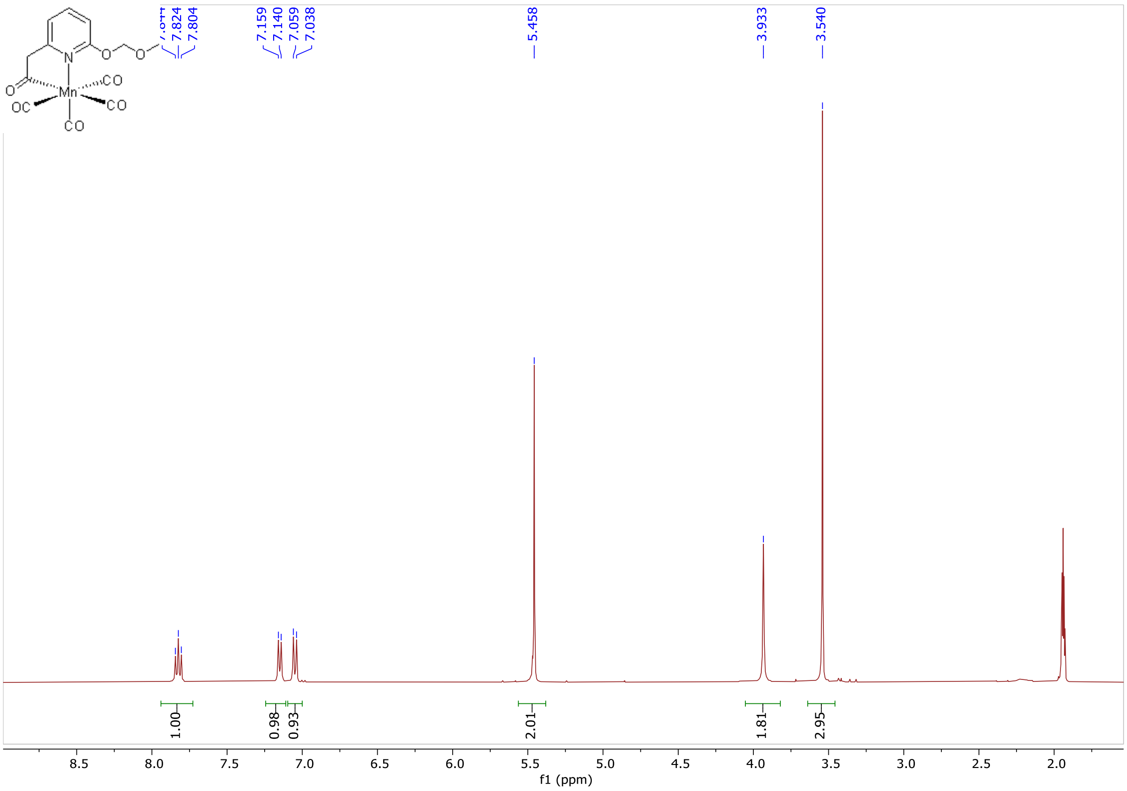

Figure S28. ^{1}H NMR spectrum of complex **1g**

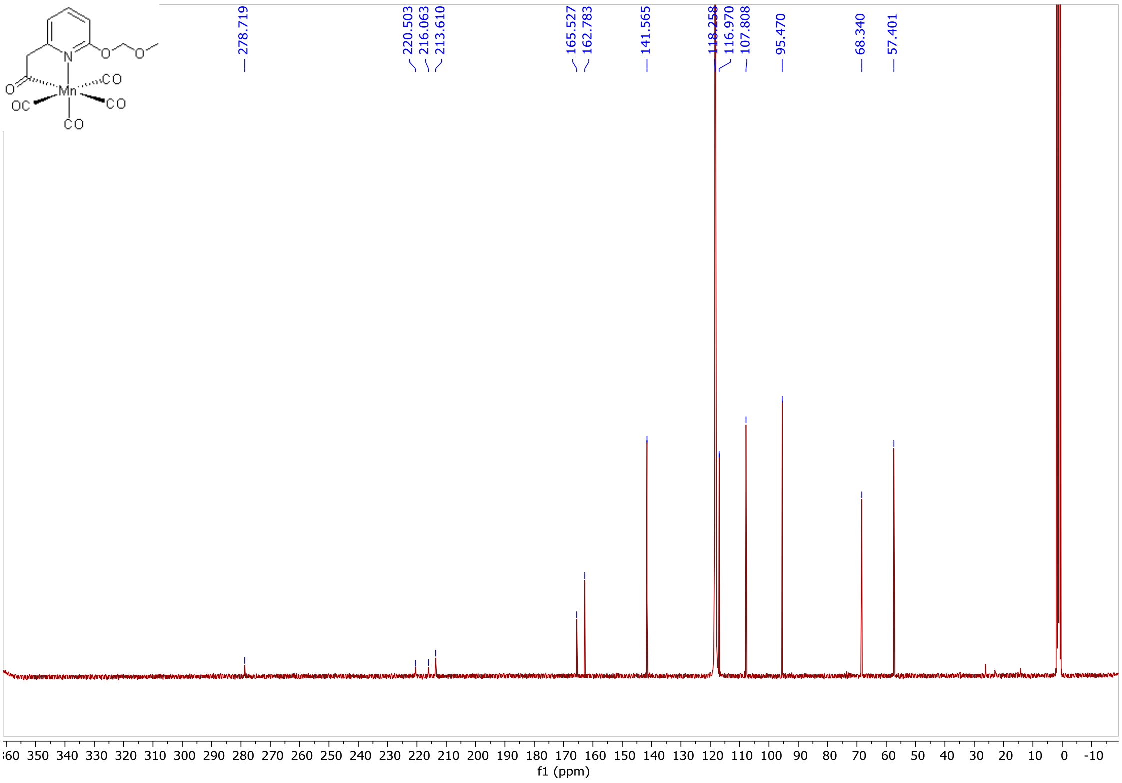

Figure S29. ^{13}C NMR spectrum of complex **1g**

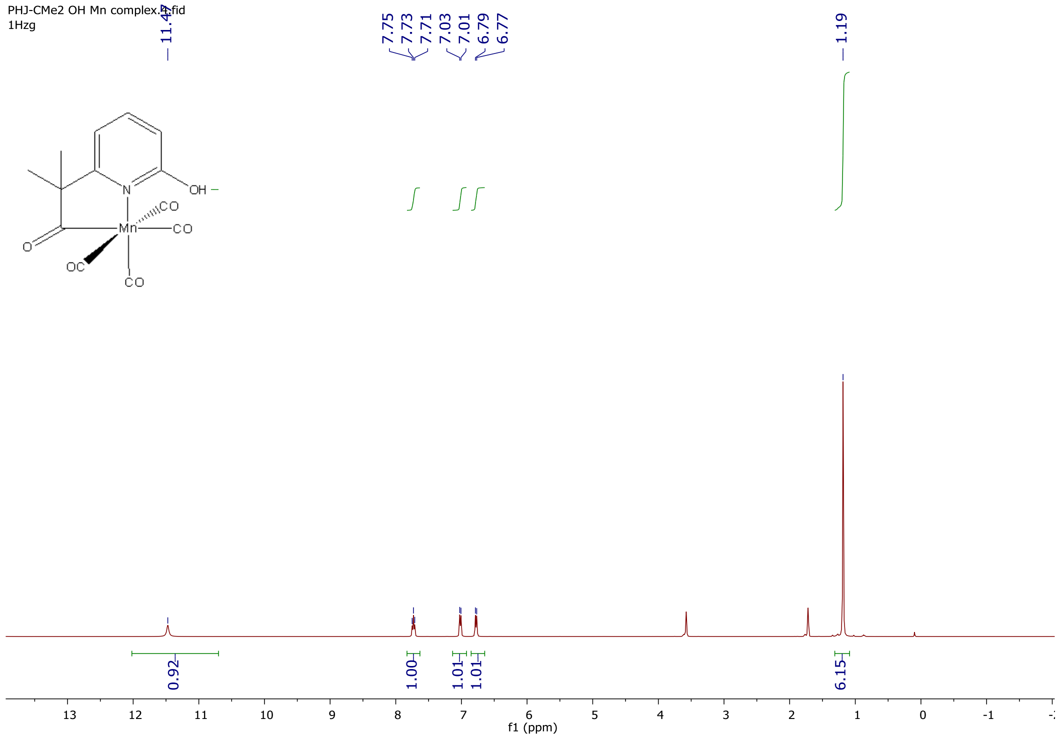

Figure S30. ^{1}H NMR spectrum of complex **1b**

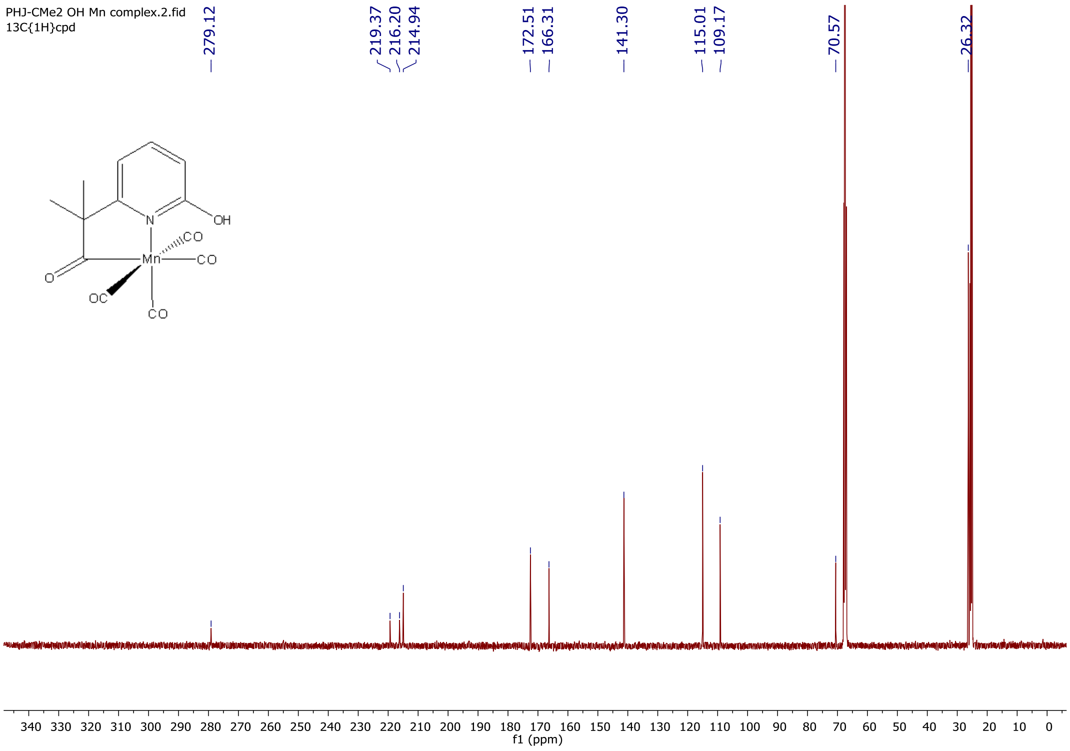

Figure S31. ^{13}C NMR spectrum of complex **1b**

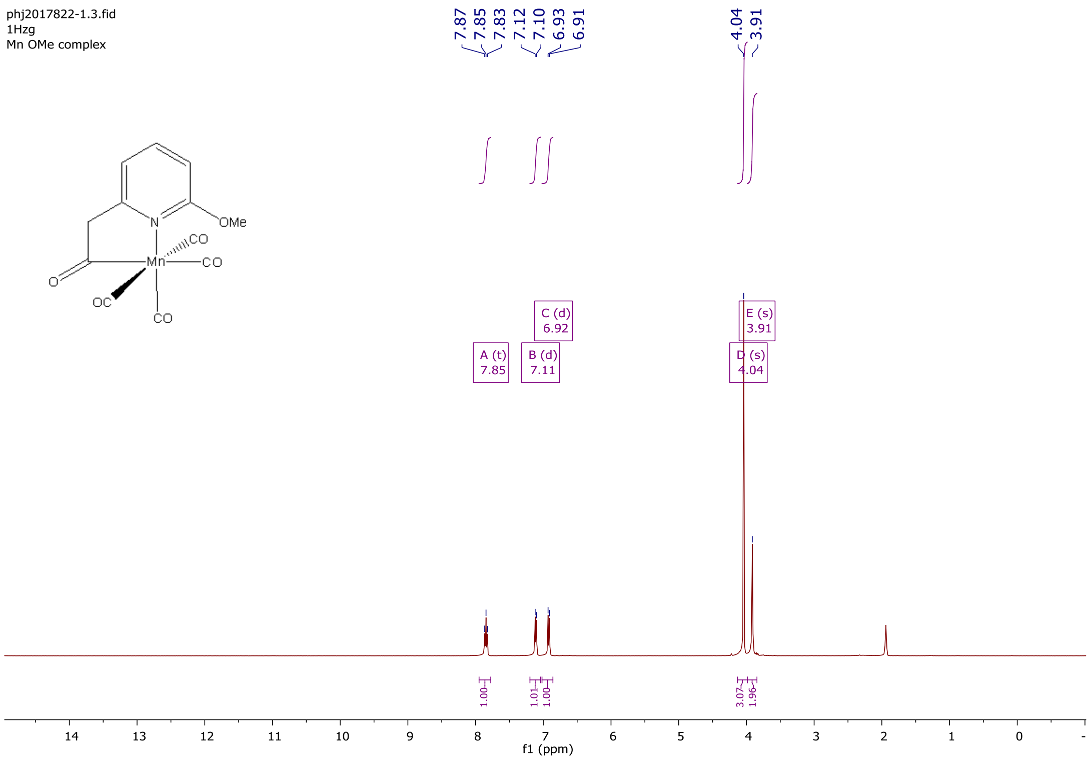

Figure S32. ^{1}H NMR spectrum of complex **1c**

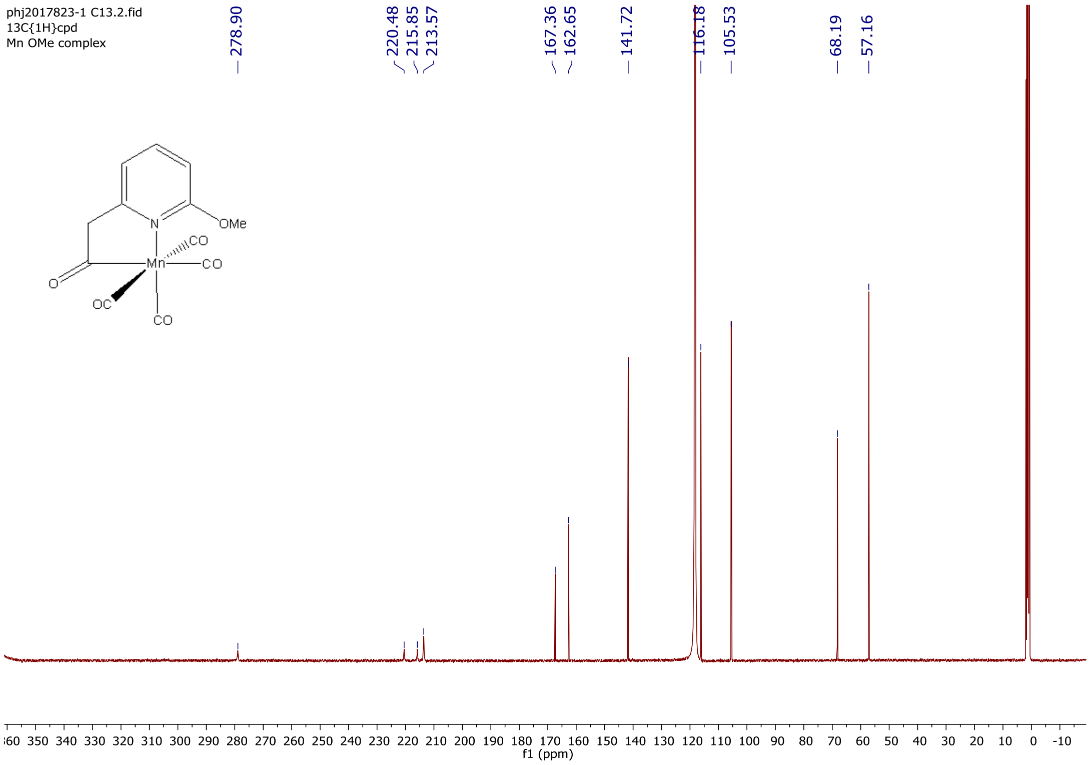

Figure S33. ^{13}C NMR spectrum of complex **1c**

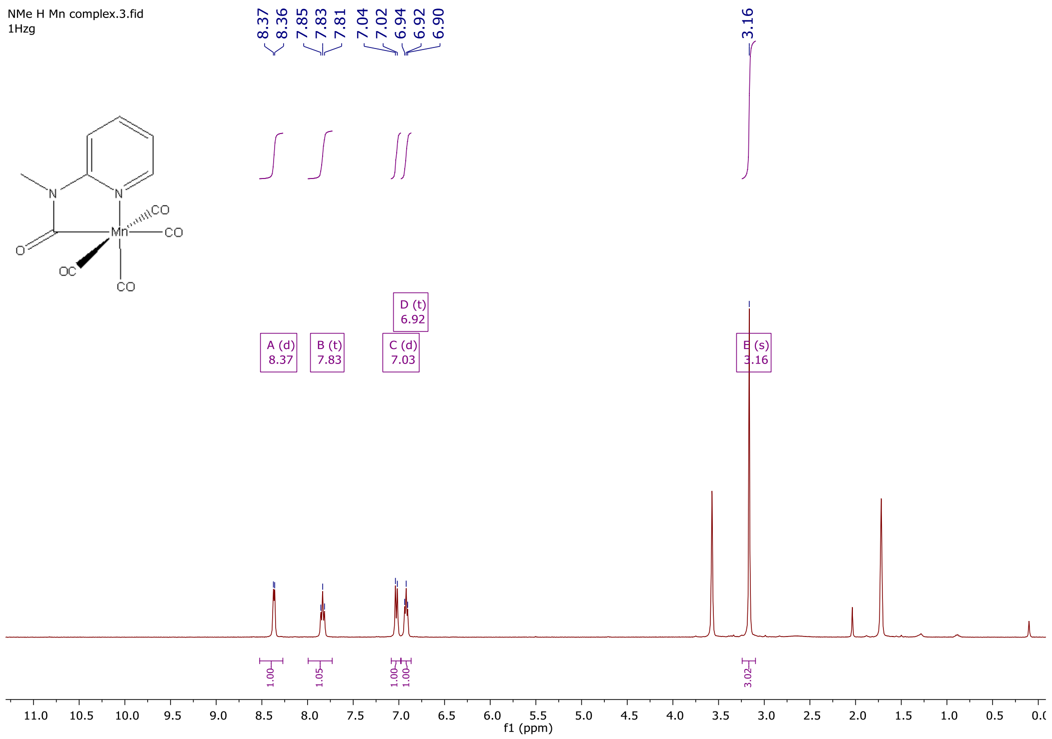

Figure S34. ^{1}H NMR spectrum of complex **1d**

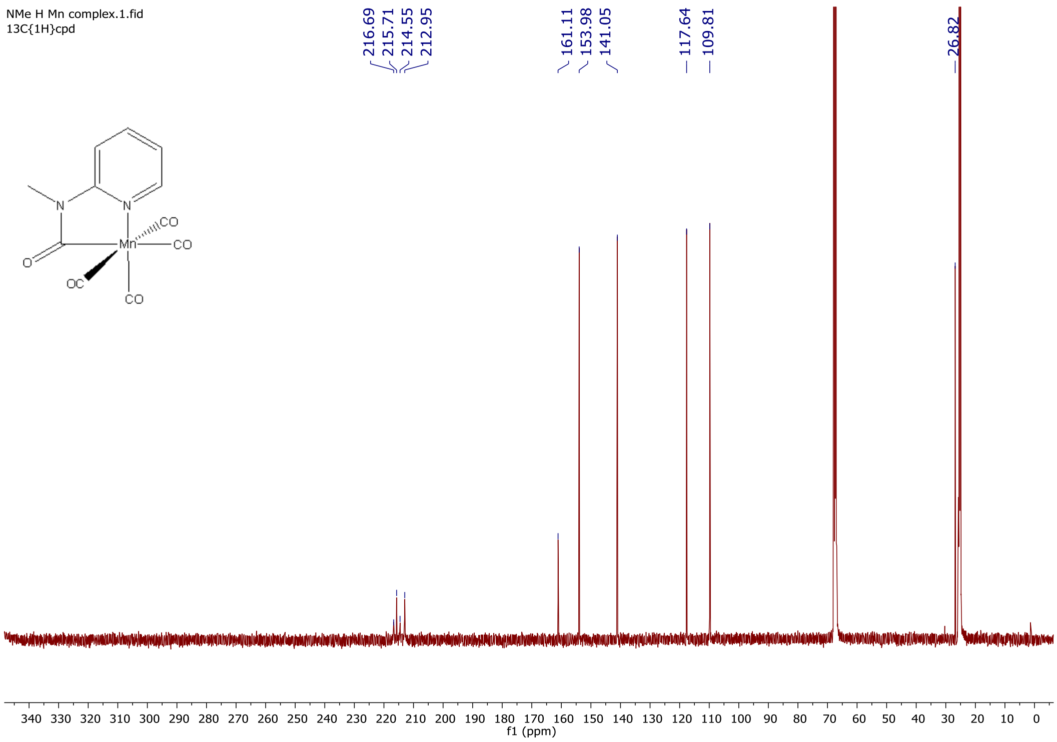

Figure S35. ^{13}C NMR spectrum of complex **1d**

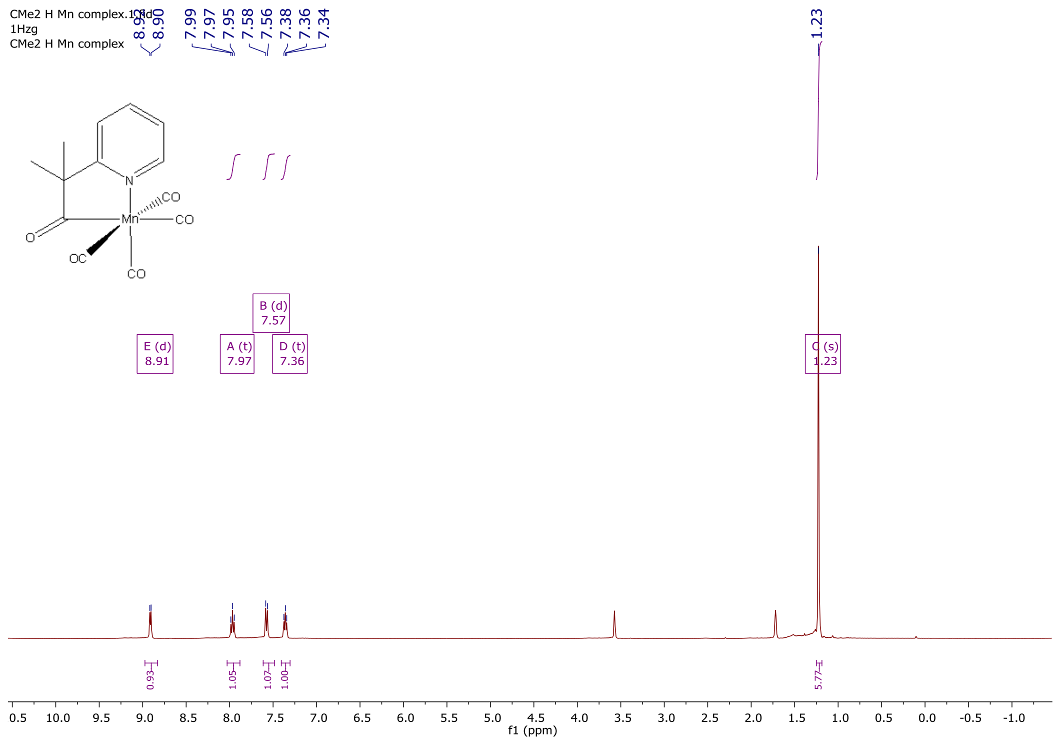

Figure S36. ^{1}H NMR spectrum of complex **1e**

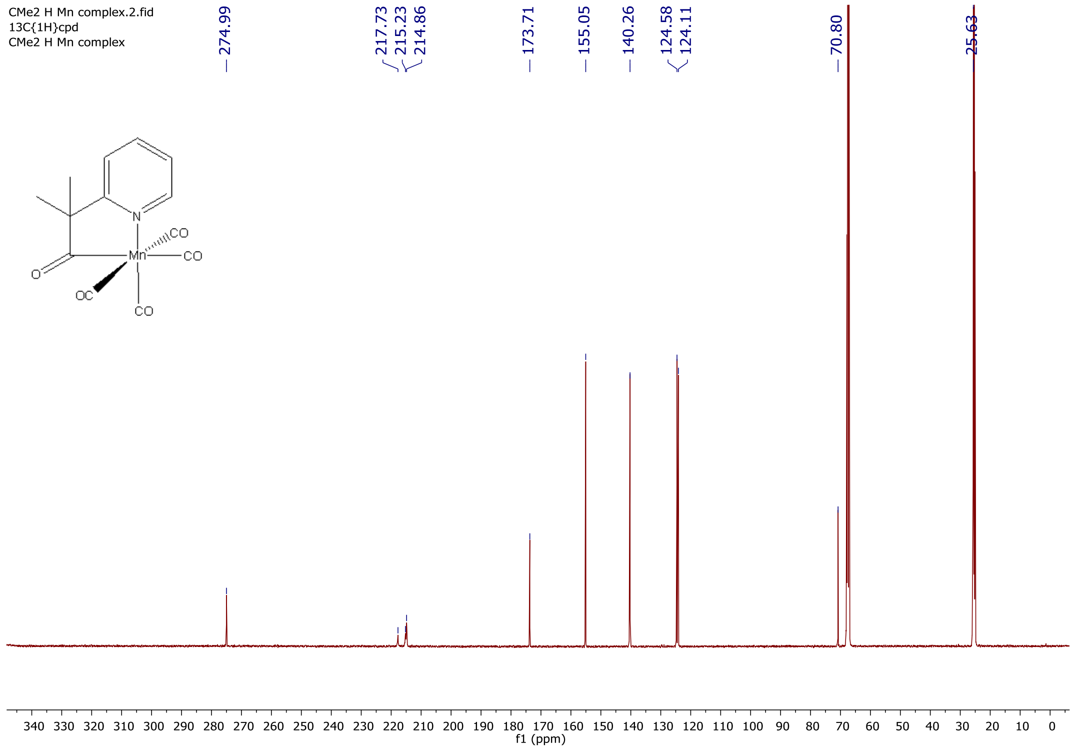

Figure S37. ^{13}C NMR spectrum of complex **1e**

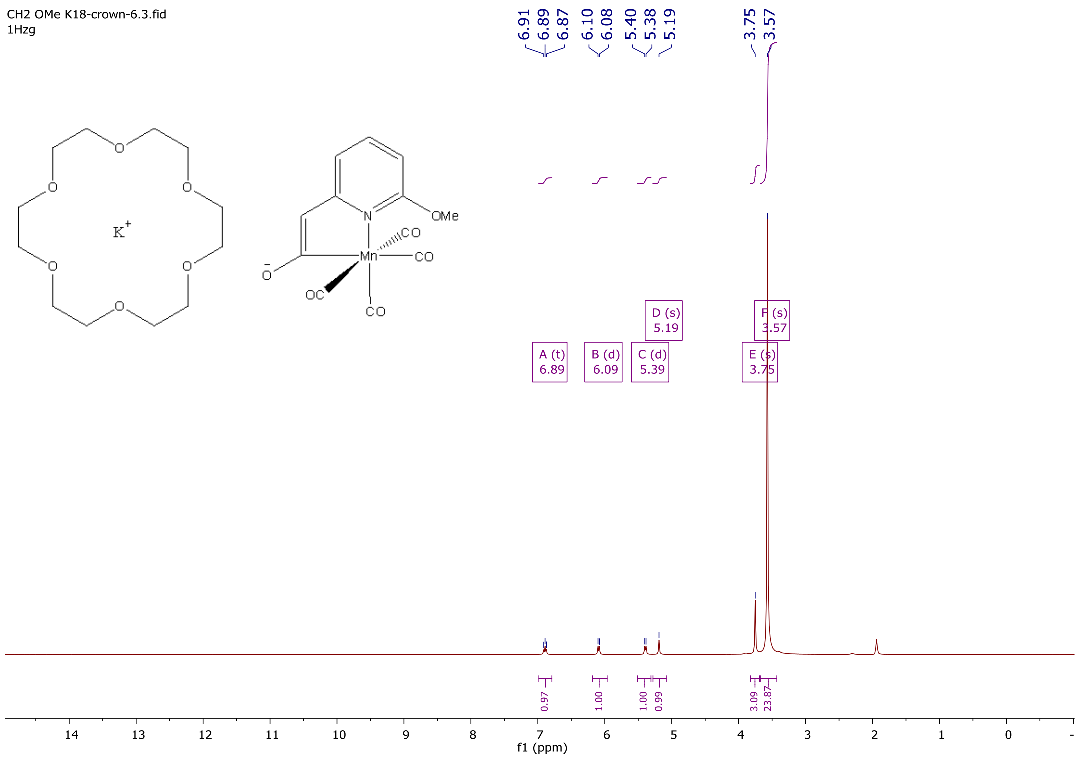

Figure S38. ^{1}H NMR spectrum of complex **3**(18-crown-6)

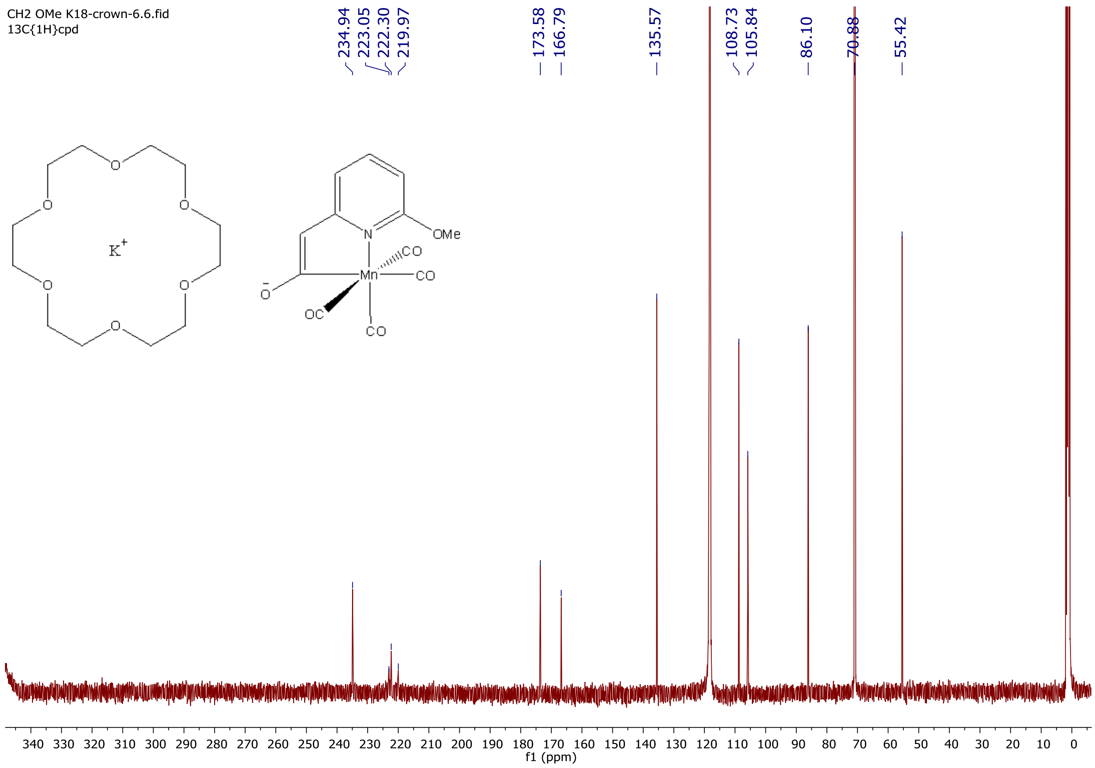

Figure S39. ^{13}C NMR spectrum of complex **3**(18-crown-6)

1.10 Cyclic voltammetric data of the Mn complexes

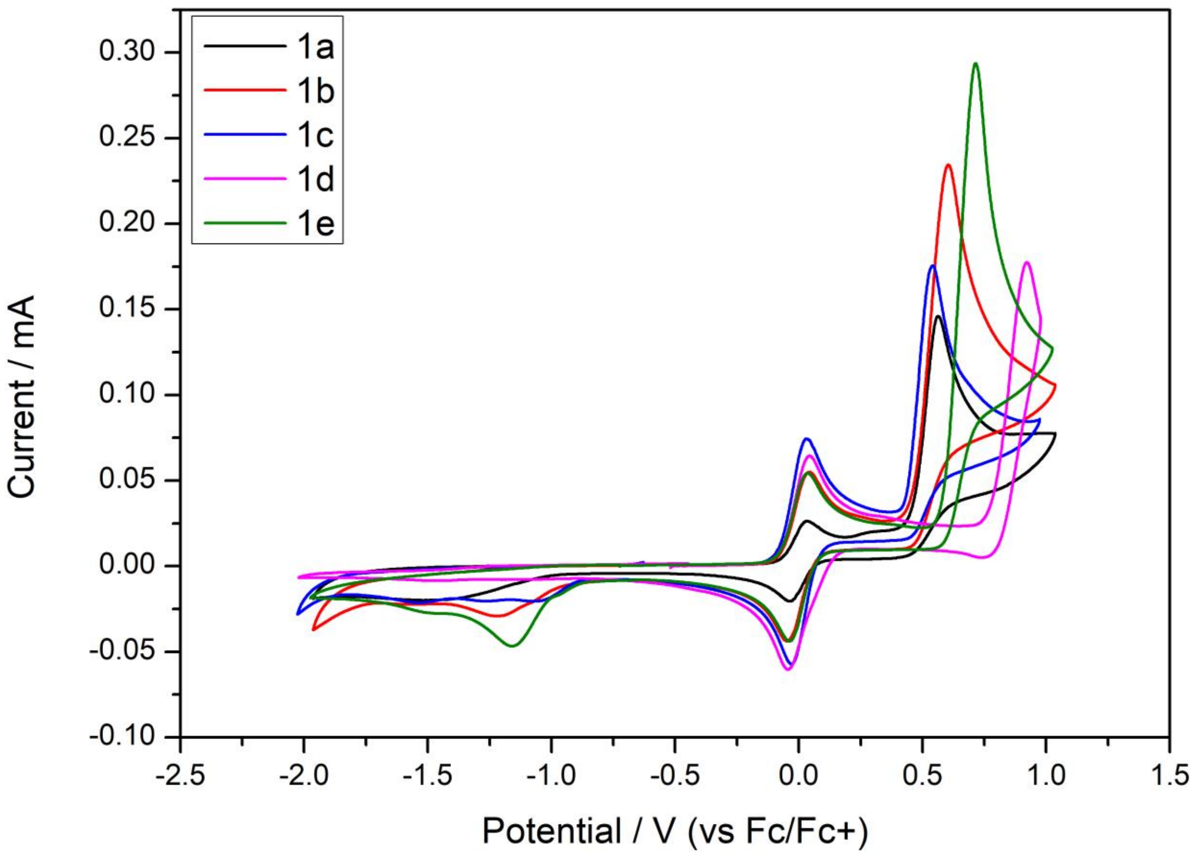

Figure S40. Cyclic voltammograms of complexes **1a-1e**. Conditions: 5 mM of complex dissolved in CH_3CN; electrolyte: 100 mM Bu_4NBF_4; scan rate: 100 mV/S; working electrode: glassy carbon (d = 3 mm); counter electrode: platinum wire; reference electrode: Ag|AgCl, KCl. Ferrocene (3 mM) was used as an internal reference. The reversible peaks near 0 V were due to ferrocene.

2. Supporting information for enzymatic study

2.1 IR spectra of [Mn]-hydrogenases and [Fe]-hydrogenase

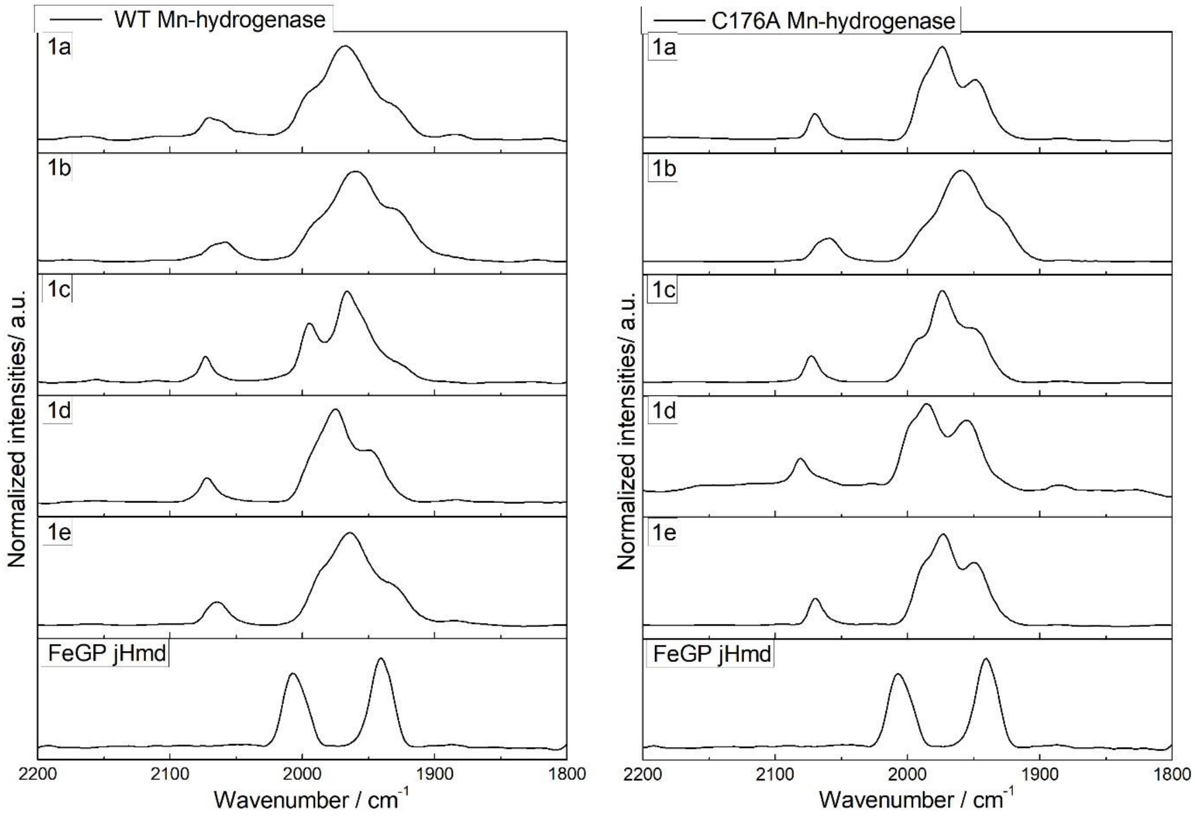

Figure S41. IR spectra of CO region (2200 ~ 1800 cm^{-1}) of jHmd Mn hydrogenases for wild type (left) and C176A mutant (right), respectively, with various Mn cofactors (**1a-e**). IR spectra of wild type jHmd with FeGP cofactor are presented at the bottom of each figures as comparison.

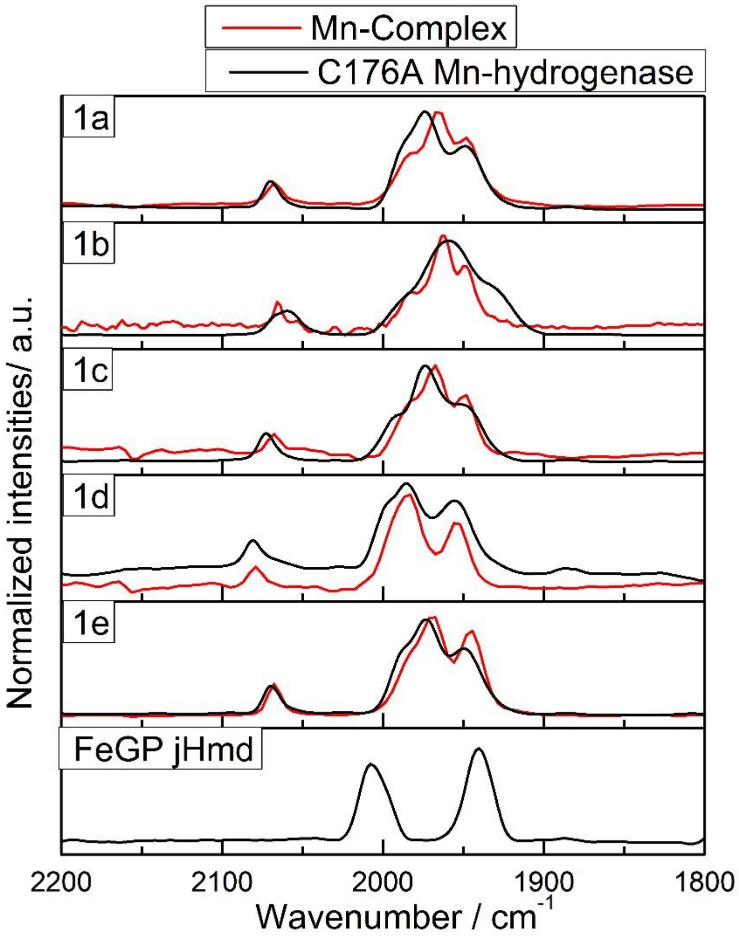

Figure S42 A comparison of the IR spectra of jHmd(C176A) Mn-hydrogenases (black) with those of the corresponding Mn complexes (red). IR spectra of wild type jHmd with FeGP cofactor are presented at the bottom of each figures as comparison.

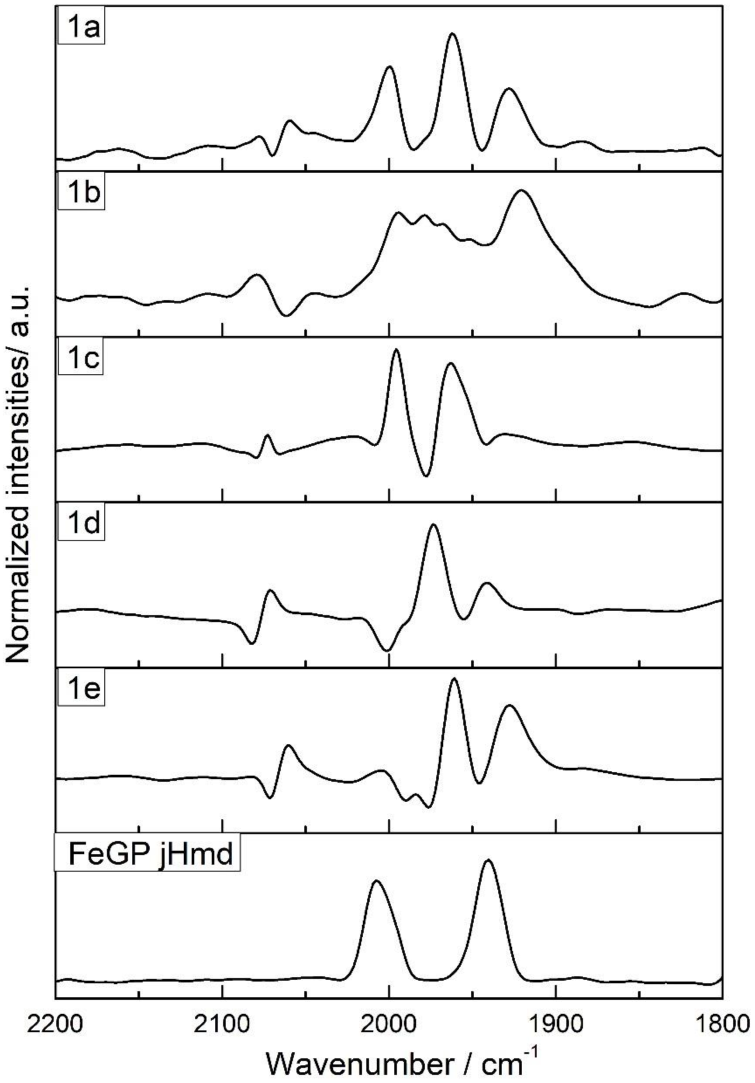

Figure S43 Subtracted IR spectra. The IR spectra of the WT jHmd Mn-hydrogenases are subtracted by the IR spectra of the corresponding C176A jHmd Mn-hydrogenases. The remaining CO peaks are expected to come from the specifically bound Mn complexes. IR spectra of wild type jHmd with FeGP cofactor are presented at the bottom of each figures as comparison.

2.2 Calculation methods for occupancy

jHmd (C176A) enzymes are assumed to have no specific bonding complex as sulfur ligand from Cys176 was removed. However, their IR spectra still exhibit strong CO peaks in the range of 1930 to 2100 cm^{-1} (Figure S41, right). These peaks come from non-specifically bound Mn complexes. The small peak at around 2070 cm^{-1} was characteristic as it was at the same place as that of its Mn complex precursor (Figure S42). The IR spectra of jHmd (wild) enzymes are expected to be a mixture of CO peaks originated from both specifically and non-specifically bound Mn complexes (Figure S41, left).The IR spectra of jHmd (wild)-FeGP sample, on the other hand, has no peaks at around 2070 cm^{-1}. Therefore, we attributed CO peak at around 2070 cm^{-1} only to non-specifically bound complex in both jHmd (wild) enzymes and jHmd (C176A) enzymes. The actual peak area contributed by a specifically bound Mn complex in a jHmd (wild) enzyme can be calculated by deducting the peak area from non-specifically bound Mn complexes.

Based on the analysis above, occupancy rate of jHmd (wild) enzymes by specifically bound complex can be calculated. CO bands of [Mn]-hydrogenases consist of a small peak around 2070 cm^{-1} and a large feature overlapping of three bands between 1930 and 1995 cm^{-1}. We tentatively call these features for

the former as "CO(high)" and the latter as "CO(low)". The $CO(low)_{jHmd\ (C176A)\text{-}\mathbf{1}}/CO(high)_{jHmd\ (C176A)\text{-}\mathbf{1}}$ ratio (k) of a jHmd (C176A) [Mn]-hydrogenase represents the CO(low)/CO(high) ratio of its corresponding non-specifically bound complex. This ratio is expected to be the same in both jHmd (wild) enzymes and jHmd (C176A) enzymes for their non-specifically bound complex. While in a jHmd (wild) enzyme, the existence of specifically bound complex make the $CO(low)_{jHmd\ (wide)\text{-}\mathbf{1}}/CO(high)_{jHmd\ (wide)\text{-}\mathbf{1}}$ ratio bigger than k. As shown in Table S4, the CO(low)/CO(high) ratios remain consistent among different [Mn]-hydrogenase samples reconstituted from the same Mn complex. The CO(low)/CO(high) ratios of jHmd (wild) type [Mn]-hydrogenase are always higher than that of jHmd (C176A) [Mn]-hydrogenases reconstituted from the same Mn complexes, supporting our hypothesis. The CO peak area contributed by specifically bound complex is then calculated using equation 2 in scheme S2. Thus, occupancy rate (Table 2 and Table S5) of a specifically bound Mn complex can be calculated by comparing specifically bound CO peak area to amide band II peak area ratio ($R_{jHmd\ (wild)\text{-}\mathbf{1}}$) with that of wide type [Fe]-hydrogenase (R_{FeGP}) which is set to have a 100% occupancy (equation 5, Scheme S2). It is possible that a 100% occupancy of a Mn complex will give a different CO peak area to wide type [Fe]-hydrogenase. However, a systematic correction to the resulting occupancies will not change the relative rates of different variants of [Mn]-hydrogenase.

$$k = \frac{CO(low)_{jHmd\ (C176A)\text{-}\mathbf{1}}}{CO(high)_{jHmd\ (C176A)\text{-}\mathbf{1}}} \qquad (1)$$

$$CO(specific)_{jHmd\ (wild)\text{-}\mathbf{1}} = CO(low)_{jHmd\ (wild)\text{-}\mathbf{1}} - k \times CO(high)_{jHmd\ (wild)\text{-}\mathbf{1}} \qquad (2)$$

$$R_{jHmd\ (wild)\text{-}\mathbf{1}} = \frac{CO(specific)_{jHmd\ (wild)\text{-}\mathbf{1}}}{\text{amide band II}_{jHmd\ (wild)\text{-}\mathbf{1}}} \qquad (3)$$

$$R_{FeGP} = \frac{CO_{jHmd\ (wild)\text{-}FeGP}}{\text{Amide band II}_{jHmd\ (wild)\text{-}FeGP}} \qquad (4)$$

$$\text{Occupancy rate} = \frac{R_{jHmd\ (wild)\text{-}\mathbf{1}}}{R_{FeGP}} \times 100\% \qquad (5)$$

$CO(low)_{jHmd\ (C176A)\text{-}\mathbf{1}}$	=	CO(low) peak area of jHmd (C176A)-**1** sample
$CO(high)_{jHmd\ (C176A)\text{-}\mathbf{1}}$	=	CO(high) peak area of jHmd (C176A)-**1** sample
$CO(low)_{jHmd\ (wide)\text{-}\mathbf{1}}$	=	CO(low) peak area of jHmd (wide)-**1** sample
$CO(high)_{jHmd\ (wide)\text{-}\mathbf{1}}$	=	CO(high) peak area of jHmd (wide)-**1** sample
$CO(specific)_{jHmd\ (wild)\text{-}\mathbf{1}}$	=	CO peak area contributed from specifically bound Mn complex in a jHmd (wide)-**1** sample
$CO_{jHmd\ (wild)\text{-}FeGP}$	=	Total CO(high) peak area of jHmd (wide)-FeGP sample
$\text{Amide band II}_{jHmd\ (wild)\text{-}FeGP}$	=	Amide II peak area of jHmd (wide)-FeGP sample

Scheme S2. Equations related to the calculation of occupancy rate of [Mn]-hydrogenase samples

Table S4. Integrated intensities of CO peak areas from IR data and corresponding CO(low)/CO(high) ratio.

Entry	Sample number	Amide band II area	Peak area of CO(low)	Peak area of CO(high)	CO(low)/CO(high) ratio
1	jHmd (wild)-**1a**-1	61864	818	72	11.4
2	jHmd (wild)-**1a**-2	20443	391	35	11.2
3	average				11.3
4	jHmd (wild)-**1b**-1	21555	242	25	9.7
5	jHmd (wild)-**1b**-2	10852	134	14	9.6
6	jHmd (wild)-**1b**-3	27114	317	34	9.3
7	jHmd (wild)-**1b**-4	14299	147	15	9.8
8	average				9.6
9	jHmd (wild)-**1c**-1	16696	232	27	8.6
10	jHmd (wild)-**1c**-2	10045	156	18	8.7
11	jHmd (wild)-**1c**-3	11878	212	26	8.2
12	jHmd (wild)-**1c**-4	10427	139	17	8.2
13	average				8.4
14	jHmd (wild)-**1d**-1	31734	262	24	10.9
15	jHmd (wild)-**1d**-2	30313	234	22	10.6
16	jHmd (wild)-**1d**-3	35467	288	27	10.7
17	jHmd (wild)-**1d**-4	25950	226	21	10.8
18	average				10.7
19	jHmd (wild)-**1e**-1	27635	180	11	16.4
20	jHmd (wild)-**1e**-2	43728	343	17	20.2
21	jHmd (wild)-**1e**-3	27743	250	14	17.9
22	average				18.1
23	jHmd (C176A)-**1a**-1	--	267	25	10.7
24	jHmd (C176A)-**1a**-2	--	182	17	10.7
25	jHmd (C176A)-**1a**-3	--	358	33	10.8
26	jHmd (C176A)-**1a**-4	--	210	20	10.5
27	jHmd (C176A)-**1a**-5	--	207	19	10.9
28	average				10.7
29	jHmd (C176A)-**1b**-1	--	337	45	7.5
30	jHmd (C176A)-**1b**-2	--	334	46	7.3
31	jHmd (C176A)-**1b**-3	--	247	34	7.3
32	jHmd (C176A)-**1b**-4	--	459	62	7.4
33	jHmd (C176A)-**1b**-5	--	491	66	7.4
34	average				7.4
35	jHmd (C176A)-**1c**-1	--	356	45	7.9
36	jHmd (C176A)-**1c**-2	--	437	54	8.1
37	jHmd (C176A)-**1c**-3	--	328	44	7.5
38	jHmd (C176A)-**1c**-4	--	413	54	7.6
39	jHmd (C176A)-**1c**-5	--	515	63	8.2
40	average				7.9
41	jHmd (C176A)-**1d**-1	--	52	6	8.7
42	jHmd (C176A)-**1d**-2	--	72	7	10.3
43	jHmd (C176A)-**1d**-3	--	51	5	10.2
44	jHmd (C176A)-**1d**-4	--	77	7	11.0
45	average				10.0
46	jHmd (C176A)-**1e**-1	--	353	27	13.1
47	jHmd (C176A)-**1e**-2	--	342	26	13.2
48	jHmd (C176A)-**1e**-3	--	315	24	13.1
49	jHmd (C176A)-**1e**-4	--	322	25	12.9
50	jHmd (C176A)-**1e**-5	--	259	20	13.0
51	average				13.0
52	jHmd (wide)-FeGP-1	23119	77	--	--
53	jHmd (wide)-FeGP-2	28158	106	--	--
54	jHmd (wide)-FeGP-3	28831	107	--	--
55	jHmd (wide)-FeGP-4	15626	54	--	--
56	jHmd (wide)-FeGP-5	17618	75	--	--

Table S5. Occupation rates, specific activities and actual specific activities of semi-synthetic [Mn]-hydrogenase samples

**Entry**	**Mn-hydrogenase sample**	**Measured specific**	**Measured specific**	**Occupancy**	**actual Specific activity**	**actual Specific activity**

		activity forward	activity reverse		forward	reverse
1	jHmd (wild)-**1a**-1	1.65	0.30	20.0%	8.27	1.50
2	jHmd (wild)-**1a**-2	1.50	0.10	20.6%	7.28	0.49
3	**average**			**20.3 ± 0.5%**	**7.8 ± 0.7**	**1.0 ± 0.7**
4	jHmd (wild)-**1b**-1	0.99	ND	72.3%	1.37	ND
5	jHmd (wild)-**1b**-2	0.80	ND	76.6%	1.04	ND
6	jHmd (wild)-**1b**-3	0.65	ND	66.1%	0.98	ND
7	jHmd (wild)-**1b**-4	0.72	ND	68.8%	1.05	ND
8	**average**			70.9 ± 4.6%	1.1 ± 0.17	ND
9	jHmd (wild)-**1c**-1	7.78	8.78	32.2%	24.20	27.31
10	jHmd (wild)-**1c**-2	9.43	8.26	39.2%	24.05	21.07
11	jHmd (wild)-**1c**-3	6.84	7.75	17.6%	38.89	44.06
12	jHmd (wild)-**1c**-4	8.62	7.88	14.1%	61.15	55.90
13	**average**[a]			25.8 ± 11.9%	37 ± 17	37 ± 16
14	jHmd (wild)-**1d**-1	1.44	ND	17.9%	8.03	ND
15	jHmd (wild)-**1d**-2	1.30	ND	11.7%	11.09	ND
16	jHmd (wild)-**1d**-3	1.30	ND	12.9%	10.06	ND
17	jHmd (wild)-**1d**-4	1.24	ND	15.8%	7.84	ND
18	**average**			14.6 ± 2.8%	9.3 ± 1.6	ND
19	jHmd (wild)-**1e**-1	ND	ND	35.8%	ND	ND
20	jHmd (wild)-**1e**-2	ND	ND	74.9%	ND	ND
21	jHmd (wild)-**1e**-3	ND	ND	65.7%	ND	ND
22	**Average**			58.8 ± 20.5%	ND	ND

[a] The error bars are bigger for jHmd (wild)-1c samples than for others. They might be due to the uncertainty of the method to calculate the occupancy, or due to varied stability of the protein in the presence of different complexes.

2.3 Enzymatic reactivity and mechanism

Reconstitution of semi-synthetic [Mn]-hydrogenase

Reconstitution experiments was performed as previous report [3]. Mn mimics were used within 2 weeks after the synthesis of mimics and were stored at −20 °C under N_2 atmosphere before usage. Complexes were dissolved in a solution consists of 99% methanol and 1% acetic acid. The reconstitution was performed in an anoxic tent under 95%N_2/5%H_2 at 8 °C. The 2-ml reconstitution system contained 0.5-mM complex, 0.25 mM apoenzyme, 2 mM GMP and 100-mM sodium acetate buffer (pH 5.6). The mixture was incubated on ice for 1 h under anoxic conditions. Then wash-treatment was performed to remove the unbound complex using 10-mM 3-(N-morpholino)propanesulfonic acid (MOPS) /KOH (pH 7.0) containing 2 mM dithiothreitol via a 30-kDa cut-off ultrafilter with at least totally 1000 fold dilution. Finally, the reconstituted holoenzyme was concentrated to ~50 mg/ml. The prepared semi-synthetic [Mn]-hydrogenase was quickly frozen in liquid N_2 and stored at −75 °C.

Enzymatic activity assay semi-synthetic [Mn]-hydrogenase

Measurements were performed as previous report[3]. Hydrogenation of methenyl-H_4MPT^+ to methylene-H_4MPT (forward direction) and dehydrogenation of methylene-H_4MPT to methenyl-H_4MPT^+ (reverse direction) were used for enzymatic activity assay. For the forward direction, 20 µM methenyl-H_4MPT^+ (final concentration) was injected into a 0.7 ml reaction system containing 120 mM potassium phosphate buffer pH 7.5 and 1 mM EDTA under 100% H_2 gas phase at 40 °C. The reaction was started by adding 10 µl of reconstituted semisynthetic enzyme and detected by recording the decrease of the absorbance at 336 nm. For the reverse direction, 20 µM methylene-H_4MPT (final concentration) was injected into a 0.7 ml reaction system that contained 120 mM potassium phosphate buffer pH 6.0 containing 1 mM EDTA under a 100% N_2 gas phase at 40 °C. The reaction was started by adding 10 µl of the reconstituted semisynthetic enzyme and detected by recording the increase of the absorbance at 336 nm. The activities were calculated with the extinction coefficient of methenyl-H_4MPT^+ ($\varepsilon_{336\ nm}$ = 21.6 mM^{-1} cm^{-1}) [10]. One unit (U) of enzymatic activity was defined as the amount

of enzyme that catalyses a decrease of 1 μmol min^{-1} methenyl-H_4MPT^+ (forward direction) or an increase of methenyl-H_4MPT^+ (reversed direction).

Table S6. TOFs of [Mn]-hydrogenases in enzymatic reactions and estimated TOFs of Mn complexes in H_2/D_2 exchange experiments[1].

Entry	Samples	Actual specific activity (U mg^{-1}) (forward)	TOF(s^{-1})	Actual specific activity (U mg^{-1}) (reverse)	TOF(s^{-1})
1	jHmd (wild)-**1a**	7.8 ± 0.7	4.9 ± 0.4	1.0 ± 0.7	0.6 ± 0.4
2	jHmd (wild)-**1b**	1.1 ± 0.15	0.7 ± 0.1	ND	
3	jHmd (wild)-**1c**	37 ± 17	24 ± 11	37 ± 16	24 ± 10
4	jHmd (wild)-**1d**	9.3 ± 1.6	5.9 ± 1.0	ND	
5	jHmd (wild)-**1e**	ND		ND	
6	jHmd (H14A)-**1a**	ND	--	ND	--
7	jHmd (H14A)-**1b**	ND	--	ND	--
8	jHmd (H14A)-**1c**	ND	--	ND	--
9	jHmd (H14A)-**1d**	0.5 ± 0.2	--	ND	--
10	jHmd (H14A)-**1e**	ND	--	ND	--
11	jHmd (C176A)-**1a**	0.06 ± 0.07	--	ND	--
12	jHmd (C176A)-**1b**	ND	--	ND	--
13	jHmd (C176A)-**1c**	ND	--	ND	--
14	jHmd (C176A)-**1d**	ND	--	ND	--
15	jHmd (C176A)-**1e**	ND	--	ND	--
16	jHmd (wild)-FeGP	410 ± 12	260 ± 8	360 ± 16	230 ± 10

[1] The volume of NMR tube is 1.7 mL. According to PV=nRT, there was 1.37 mmol H_2/D_2 gas molecules. The added catalyst was 0.03 mmol. In theory, if every catalyst did one turnover and H^+/D^+ was fully exchanged, we will have a HD/H_2 ratio of 1/58. If every catalyst did 10 turnover and H^+/D^+ was fully exchanged, we will have a HD/H_2 ratio around 1/6 (assuming only H_2 and D_2 molecule did reaction). If every catalyst did 20 turnover and H^+/D^+ was fully exchanged, we will have a HD/H_2 ratio around 1/2.4 (assuming only H_2 and D_2 molecule did reaction).Therefore, in most cases in the H_2/D_2 exchange experiments, there was less than 10 turnover in 21600 seconds (0.0005 s^{-1}). The highest TOF (Table 1 entry 7 in the main text) was about 20 turnovers in 600 seconds (TOF = 0.03 s^{-1}).

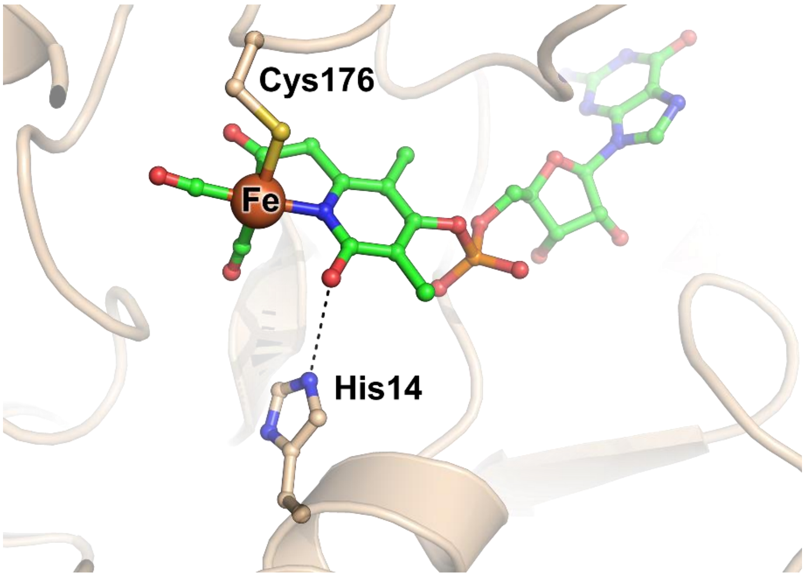

Figure S44 Hydrogen bond interaction between His 14 and 2-OH obtained from crystal structure of [Fe]-hydrogenase [11]

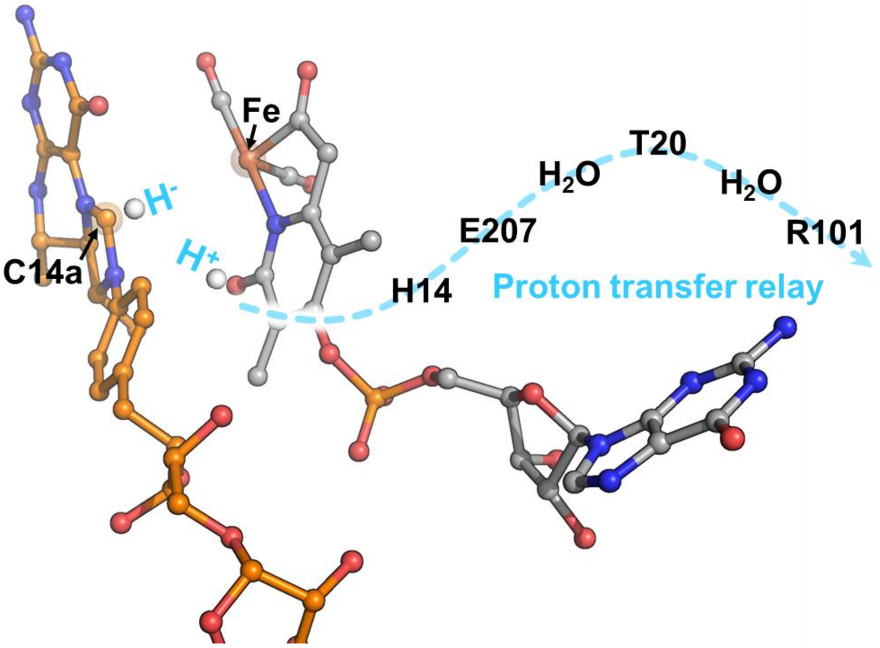

Figure S45. Proton relay process proposed in native [Fe]-hydrogenase

References

[1] H.-J. Pan, X. Hu, *Angew. Chem. Int. Ed.* **2020**, *59*, 4942-4946.

[2] J. Hou, M. Fang, A. J. P. Cardenas, W. J. Shaw, M. L. Helm, R. M. Bullock, J. A. S. Roberts, M. O'Hagan, *Energy Environ. Sci.* **2014**, *7*, 4013-4017.

[3] H.-J. Pan, G. Huang, M. D. Wodrich, F. F. Tirani, K. Ataka, S. Shima, X. Hu, *Nat. Chem.* **2019**, *11*, 669-675.

[4] **2015**.

[5] A. J. M. K.-B. Duisenberg, L. M. J.; Schreurs, A. M. M. , *J. Appl. Cryst.* **2003**, *36*, 220-229.

[6] R. H. Blessing, *Acta Cryst.* **1995**, *A51*, 33-38.

[7] G. M. Sheldrick, *Acta Cryst.* **2015**, *A71*, 3-8.

[8] G. M. Sheldrick, *Acta Cryst.* **2015**, *C71*, 3-8.

[9] B. Dolomanov OV, L. J., Gildea, R. J., Howard, J. A. K., Puschmann, H. , *J. Appl. Cryst.* **2009**, *42*, 339-341.

[10] C. Zirngibl, W. Van Dongen, B. Schworer, R. Von Bunau, M. Richter, A. Klein, R. K. Thauer, *Eur J Biochem* **1992**, *208*, 511-520.

[11] G. Huang, T. Wagner, M. D. Wodrich, K. Ataka, E. Bill, U. Ermler, X. Hu, S. Shima, *Nat. Catal.* **2019**, *2*, 537-543.
